# Disease and disparity in China: a view from stroke and MI disease

**DOI:** 10.1186/s12939-019-0986-2

**Published:** 2019-06-11

**Authors:** Yao Yao, Gordon Liu, Linhong Wang, Hanqing Zhao, Zhenping Zhao, Mei Zhang, Meijiao Wang, Limin Wang

**Affiliations:** 1 National School of Development, Peking University, 5 Yihe Road, Haidian District, Beijing, 100871 China; 2 China Hospital Development Institute, Shanghai Jiao Tong University, Shanghai, 200025 China; 3 China Center for Health Economic Research, Peking University, Beijing, 100871 China; 4 National Center for Chronic and Non-communicable Disease Control and Prevention, Chinese Center for Disease Control and Prevention, No.27 Nanwei Road, Xicheng District, Beijing, 100050 China

**Keywords:** Stroke, MI, Prevalence, Inequality, Social economic status

## Abstract

**Background:**

The actual distribution of stroke and myocardial infarction (MI) associated with social economic status (SES) among the Chinese population is unclear. We aim to understand the development of disparity in stroke and myocardial infarction (MI) across different income groups in Chinese population.

**Methods:**

Data about stroke and MI disease, income, gender, and areas were obtained from China Chronic Disease and Risk Factor (CCDRF) Survey in 2007, 2010, and 2013. Respondents were categorized into different income groups according to their income rank, disease rate was calculated in each group, and difference in disparities between genders, health behaviors, and areas were further identified. Association of disease prevalence rate and income was verified by logistic regression. Trends in stroke and MI disease prevalence rate across income gradients; trends in the correlation between stroke and MI disease prevalence rate and income over time; variation in stroke and MI disease levels and its disparity across income groups by gender, region, and health behavior. Disease prevalence rate is age-adjusted by using China census 2010 population structure as a standard.

**Results:**

Three waves of survey were included, the sample size in each wave was 45,095 (year 2007), 84,117 (year 2010), and 134,962 (year 2013). Four major findings were delivered. First, the stroke and MI prevalence rate of Chinese population increased from 2007 to 2013. Second, for each survey wave, a negative correlation between stroke and MI risk with income was identified, and this correlation became weaker over time. The gap in stroke and MI prevalence rate between the richest people and the poorest people decreased from 2007 (gap = 2.5 percentage points) to 2013 (gap = 1.6 percentage points). Third, the identified health inequality varied across genders, regions, and health behaviors. For example, female population used to face a sharper decline in prevalence rate when income grew, this correlation, however, faded over time. The rural-urban difference in disease risk was found to be the largest in the bottom income group (in 2013, the prevalence rate in urban area was 5%, which was 1.8% higher than rural places), this rural-urban difference converged as income increased. Fourth, conditioning on the smoking behavior, the negative association of income and stroke and MI prevalence rate was identified, however, conditioning on the drinking behavior, the association of income and disease morbidity was inconclusive.

**Conclusion:**

During 2007 and 2013, the Chinese residents experienced a growth in stroke and MI prevalence rate, meanwhile, the increase in income was associated with a decrease in prevalence rate. However, this health disparity became weaker over time since the prevalence rate was more equally distributed across income gradients as time passed by. Although male population faced a systematically higher stroke and MI disease risk than female, the prevalence disparity in different income groups were similar in both sexes in 2013. In addition, there were also regional differences in inequality in terms of the association of disease and income.

**Electronic supplementary material:**

The online version of this article (10.1186/s12939-019-0986-2) contains supplementary material, which is available to authorized users.

## Background

China achieved significant in economic growth after decades of development. In 2013, the gross domestic product (GDP) of China measured at purchasing power parity (PPP) reached 1622.2386 billion, which surpassed the GDP of the United States for the first time in modern history, according to World Bank database^1^ While China’s GDP remains higher than the US, the per capita GDP in China is still at a relative low level at 8123.2 USD (in current USD for year 2016) compared with countries like the United States, where per capita GDP is 57,466.8 USD. Because of income inequality in China, per capita GDP is dramatically lower in some interior regions, meaning overall development level is misleading of China’s current economic status.

In consideration of the long discussed health-wealth linkage, where income and health are argued to be positively correlated, the association between economic inequality and health disparity is also a substantial and an important research topic [1–9]. In the field of inequality research, a plenty of issues have been revealed. For example, in the U. S, the disparity in life expectancy across individual income percentiles is illustrated thoroughly and the results show that the gap in life expectancy between the wealthiest and poorest population is 14.6 years [7]. The prevalence rates of diabetes, hypertension, stroke, and lung disease in England decrease as income grows, and also England residents are much healthier than their US counterparts at all points of the socioeconomic statuses (SES) [6]. Currently, the discussion of income inequality and health disparity is much more sufficient in the United States, United Kingdom and Europe than that in China. However, for the country that owns world’s largest population and highest economic growth, an in-depth study on the correlation between income inequality and health disparity is in urgent need.

The epidemiology of China’s population is expected to approach that of developed countries, with the rise of non-communicable diseases becoming the leading cause of death^.^ 2 But given significant within-country variation in economic development, the occurrence of non-communicable disease burden is expected to vary as well across SES. Therefore, the actual distribution of chronic disease associated with SES among the Chinese population is worth exploring, as the distribution has consequences for one fifth of the world’s population.

Our study is distinguished from previous studies in two aspects: 1) Firstly, we concentrate on the inequality of stroke and MI prevalence in the income group which enriches the disease category in current discussions about health inequalities. 2) Secondly, this paper focuses on China which is seldom studied in health disparity area by employing a unique national representative data of China.

### Objectives

In this paper, we examine stroke and myocardial infraction (MI) disease, the leading cause of death among chronic diseases both in China and the world more broadly^3^, as the measure of non-communicable disease and investigated its distribution and time trend in response to household income—one of the most commonly used measurements of SES—in a nationally representative sample of China. In doing so, we sought to answer following questions: 1) What is the association between stroke and MI and household income in China? 2) What is the trend of this association over time? 3) Is there variation in disease-income relationship among genders or regions? 4) What do these gaps look like conditional on health behaviors?

## Methods

### Data

This study was conducted by the National Health and Family Planning Commission (NHFPC) Disease Control Bureau (DCB). The dataset employed in this analysis comes from the China Chronic Disease and Risk Factor (CCDRF) Survey directed by the China Center for Disease Control Chronic Disease Control Center (CDC). This survey employed a multistage stratified cluster random sampling method in investigating the chronic disease prevalence among Chinese adults, in which geographic regions, urban-rural location, socioeconomic situation, and population size were considered, more details are available in previous studies [10–12]. The random sampling survey began in 2004 and more than 30,000 individuals were surveyed. For the following waves of survey launched in 2007, 2010, and 2013, more adult individuals were included in this survey which led to the increase in random sample size. In 2013, the sample reached more than 134,962 individuals that have information on income. Data related to both chronic disease and household income is collected, and observations with missing record in income, stroke and MI, individual identification number (ID), and weights^4^ were excluded from this analysis.^5^ China census 2010 population age distribution is implemented in adjusting disease prevalence rate.

### Measures of health and health behaviors

Our analysis was based on the survey data in 2007, 2010, and 2013. For 2007, respondents’ self-report on stroke and MI condition through the question “Have you been diagnosed with stroke or MI by a rural/community level doctor or above?” and the answers were recorded as a binary variable, where one stood for having being diagnosed of stroke or MI and zero meant none. For 2010, the question was “Have you experienced a MI or stroke for the past 12 months?” and the answers were recorded as a binary variable, one represented for having experienced stroke or MI and zero meant none. For 2013, the MI and stroke relevant questions were “Have you been diagnosed with stroke or MI by a county level medical institutes or above?” and the answers were recorded as a binary variable, one represented for having being diagnosed of stroke or MI and zero meant none. The stoke and MI questions in 2010 and 2013 are not consistent with 2007, which may lead to an overestimate or underestimate in stroke and MI prevalence rate compared with the situation in 2007.^6^In order to draw a picture of stroke and MI prevalence conditional on health behaviors, smoking and alcohol consumption were also considered. People who reported ever smoking are marked as ever-smokers^7^ and people who had an excessive drinking behavior defined by CDC is identified as excessive-drinker, people who drink but without excessive drinking behaviors is categorized as seldom-drinker, people who do not drink at all is identified as non-drinker.

### Income gradient

Household income information was identified through the question “How much is your family income per year or month?” The response rates of this question were 92, 80, and 76% for 2007, 2010, and 2013, respectively. We include all the responded samples as our data in analysis. In each survey wave, we categorized income groups in two ways: first, we ranked people by household income and then split them into 10 income groups according to decile. These steps were implemented in overall, male, female, urban, rural, eastern, central, and western samples. Second, in each income decile in the overall sample, prevalence rate among age groups, genders, and locations were compared. T test was implemented in calculating the 95% confidence interval of prevalence rate in each income decile.

### Econometric approach

All analyses were weighted by appropriate sample weights and used STATA 14.0 (STATA Corp). Correlation analysis (in Additional files) between stroke and MI and income was calculated through Pearson correlation method. Regressions of stroke and MI on income was implemented through Logistic models; statistical significance was reported with thresholds of 0.1, 0.05, and 0.01; and coefficients were allowed to differ among subsamples.

## Results

### Overall distribution of stroke and MI by household income

The mean income in the 3-year pooled sample was 4123.704 (95% confidence interval 4090.488 to 4157.073) USD per household and the pooled stroke and MI prevalence rate was 1.34% (95% confidence interval 1.3 to 1.38%). The mean household income in 2007, 2010, and 2013 was 2894.2 95% confidence interval 2815.787 to 2972.723), 3600.937 (95% confidence interval 3542.011 to 3659.426), and 6178.376 (95% confidence interval 6131.751 to 6225.106) USD, respectively (See more details on income distribution in Additional file 1: Table S1. Correspondingly, the stroke and MI prevalence rates were 1.23% (95% confidence interval 1.13 to 1.38%), 0.91%(95% confidence interval 0.85 to 0.98%), and 1.899%(95% confidence interval 1.83 to 1.97%) across the three survey waves.

**Fig. 1:**
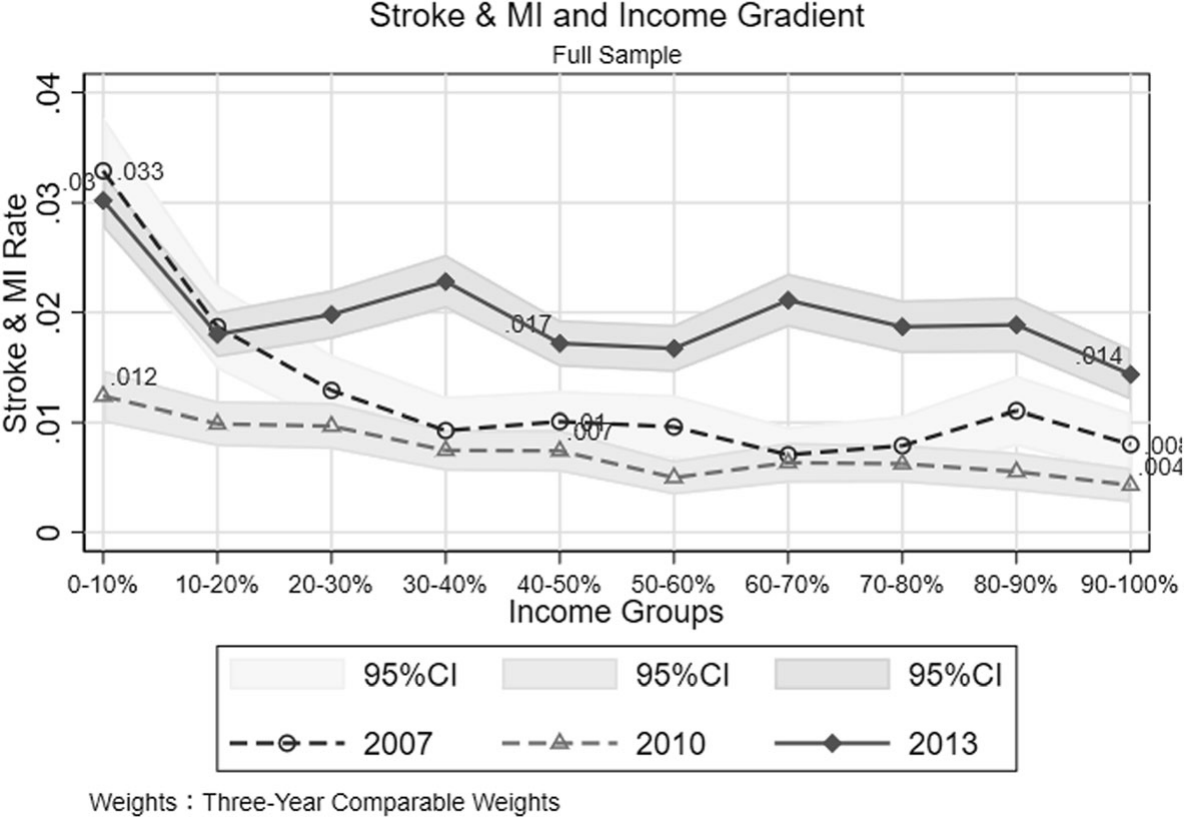
The Overall Distribution of Stroke and MI across Income Gradient

Figure 1 illustrates the overall distribution of stroke and MI by household income decile: the dashed line with hollow circle represents 2007, the dashed line with hollow triangle stand for 2010, and the solid line with solid diamond presents data from 2013. Generally, there was a significant increase in stroke and MI prevalence rate since 2007. (The 2010 stroke and MI questions were less comparable since it required that respondents should report the disease event occurred within past 12 months.) For the richest people, the difference in stroke and MI prevalence between 2013 (1.4%(95% confidence interval 1.2 to 1.67%)) and 2007 (0.8%(95% confidence interval 0.51 to 1.09%)) reached 0.6%, which means, compared with 2007, there were 6 more people with stroke and MI in every thousand residents for the wealthiest households in 2013. The increase in stroke and MI prevalence within the poorest households was smaller than that of the richest households. The downward trend in prevalence rate over income deciles, which shows the highest prevalence rate in the first decile and the lowest prevalence rate in the last decile, reveals the health inequality between rich and poor households. In the 2013 wave, however, this inequality in stroke and MI burden is partially mitigated given that the downward trend becomes less significant when the first income decile is excluded.^8^

**Fig. 2:**
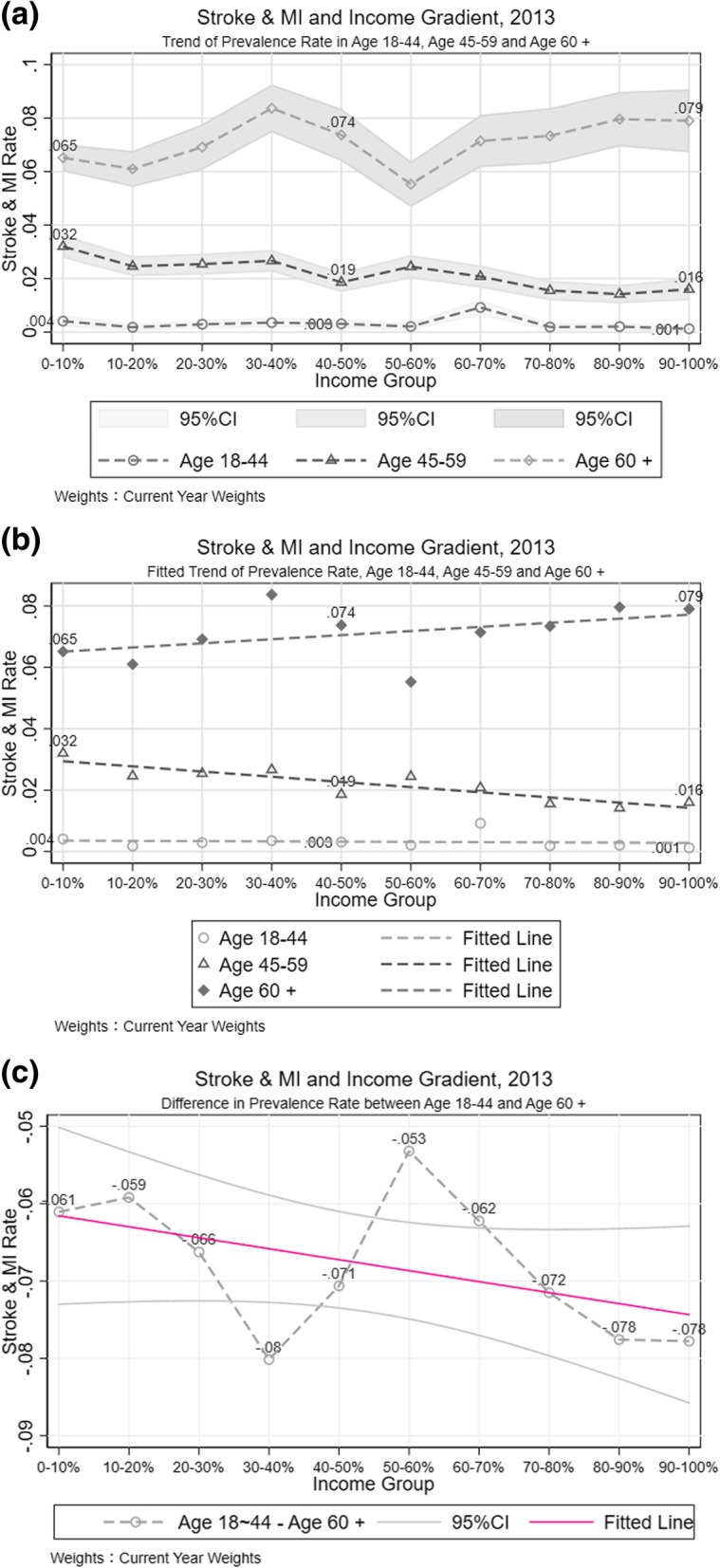
Distribution of Stroke and MI across Income Gradient, in Age Groups. **a** Trend of Prevalence Rate in Age 18-44, Age 45-59 and Age 60+. **b** Fitted Trend of Prevalence Rate, Age 18-44, Age 45-59 and Age 60+. **c** Difference in Prevalence Rate between Age 18-44 and Age 60+

Decomposing the population into three age groups in each income gradient in 2013 provides a picture where the stroke and MI prevalence rate was quite steady across income levels for people aged 18 to 44; a negative correlation between income and stroke and MI prevalence rate among people aged 45 to 59; and even though there are large fluctuations in the morbidity-income correlation among people aged 60 and above, there is a slight upward trend of stroke and MI prevalence across income categories (Fig. 2). These patterns were consistent in both 2010 and 2013 data, however, in 2007, the trend of prevalence rate in age 60 and above was not observed. By looking into the prevalence rate difference among age groups between 2010 and 2013, it is apparent that, although the extent of inequality in stroke and MI by income gradient was lower in 2013, the gaps between different age groups increased in 2013.

### Gender differences of stroke and MI by household income

**Fig. 3:**
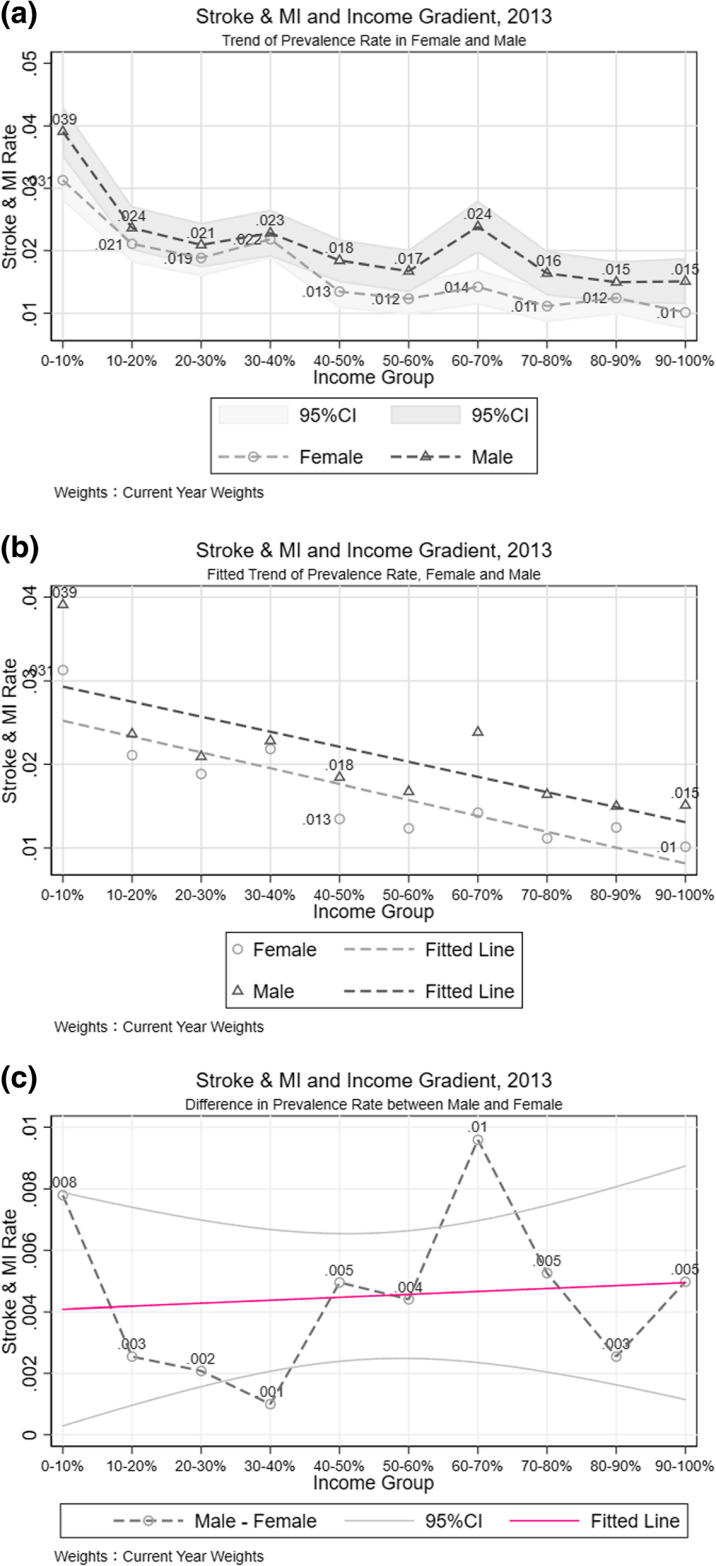
Stroke and MI Difference between Genders. **a** Trend of Prevalence Rate in Female and Male. **b** Fitted Trend of Prevalence Rate, Female and Male. **c** Difference in Prevalence Rate between Male and Female

Conditional on each income group, given the situation in 2013, there was a dramatic decline in prevalence rate as you move across income deciles (Fig. 3). By depicting the fitted line in both male and female subsamples, a different relationship between disease and income was captured in 2007 (Additional file 2: Figure S2-a). The negative correlation of stroke and MI risk with income among the female population was noticeably steeper than in the male subsample, which suggests variation in higher income’s ability to affect health outcomes. Specifically, in 2007, the trend line of prevalence rate across income groups in men was flatter than women, moving from one income decile to the next, males face a smaller decline in morbidity than females. This pattern, however, changed in 2010 (Additional file 2: Figure S2-b) and 2013 (Fig. 3-b). Compared with 2007, higher income was associated with a lower stroke and MI prevalence among males in 2010, although the relationship was still weaker than that among females. In the 2013 wave, the negative association between income and stroke and MI risk was almost parallel for males and female.

The prevalence rate disparity between genders was also observed, although it was not systematically significant across income categories, such as depicted in Fig. 3-a. In 2007 (Additional file 2: Figure S2-a), women possessed a higher stroke and MI rate in the first four income deciles, but in the highest income deciles, this relationship inverted. Plotting out the difference in stroke and MI rates between males and females, the predicted correlation line revealed an increase in inequality in stroke and MI risk between genders as income decile increased (Fig. 3-c). In 2013, the gender difference in stroke and MI prevalence is much smaller than that in 2007. The gap in prevalence rate between male and female populations, however, remained stable across income categories. The slope of predicted line in the 2013 gender difference is flatter than that in 2007, which indicates the extent to which inequality between genders across income groups mitigated: in 2013, income inequality has less of a differential impact across gender although absolutely levels of prevalence rate remain. In addition, the findings in the most recent year in our database showed that, the stroke and MI morbidity gap between females and males grows as income category increases, which revealed the fact that, in general, the stroke and MI condition is relatively equal across genders among low income households, even though men have a high prevalence rate than women. However, as income level increases, the inequality in stroke and MI prevalence rate between male and female emerges.

Given the different association of stroke and MI prevalence rate with income across male and female populations, we estimated Logistic regression models on stroke and MI prevalence. The dependent variable was the binary stroke and MI condition of respondent, and the key independent variables were different income groups. Table 1 Column [2, 3] show that, relative to the lowest income group people (0~20%), the odds ratio of having stroke and MI of women in high-income groups decreases, which means that women with high income have a lower chance of getting a stroke or MI. Specifically, the odds ratio of having stroke or MI of female cohort in the highest income group (80~100%) is 62.13% (1 − *e*^−0.971^) less than their counterparts in the lowest income group. On the other hand, male cohort in higher income percentiles does not have a statistical significant decrease in the relative risk of getting stroke or MI.

**Table 1:** Stroke and MI and Its Correlation with Income, Pooled and Subsample Analysis

	Pooled	Male	Female	Rural	Urban
Variables	[1]	[2]	[3]	[4]	[5]
Income					
20~40%	− 0.231**	− 0.0370	−0.349**	− 0.115	−0.178
	(− 2.097)	(− 0.358)	(− 2.026)	(− 0.793)	(−1.017)
40~60%	− 0.504***	− 0.253**	−0.838***	− 0.438***	−0.0904
	(− 2.827)	(− 1.963)	(− 3.469)	(− 2.592)	(− 0.375)
60~80%	− 0.238	0.119	− 0.608**	−0.309**	− 0.183
	(− 1.374)	(0.706)	(− 2.388)	(− 2.065)	(− 0.888)
80~100%	− 0.541***	− 0.165	− 0.971***	− 0.573***	− 0.304
	(− 2.739)	(− 1.021)	(− 3.584)	(− 3.922)	(− 0.884)
Weighted Obs.	1,601,683,722	878,822,001	722,529,940	1,176,985,245	424,698,477
Year	Y	Y	Y	Y	Y
Fixed Effects	Province	Province	Province	Province	Province

Notes: Robust z-statistics in parentheses. *** *p* < 0.01, ** *p* < 0.05, * *p* < 0.1. The results in table were from three-year pooled data. All regressions were controlled for Gender, Rural-Urban status, East, West, and Central regions, Drinking Behavior, Job Type, and Age Groups. Logistic regression results are expressed in the form of natural logarithm odds ratio

### Urban-rural difference of stroke and MI by household income

**Fig. 4:**
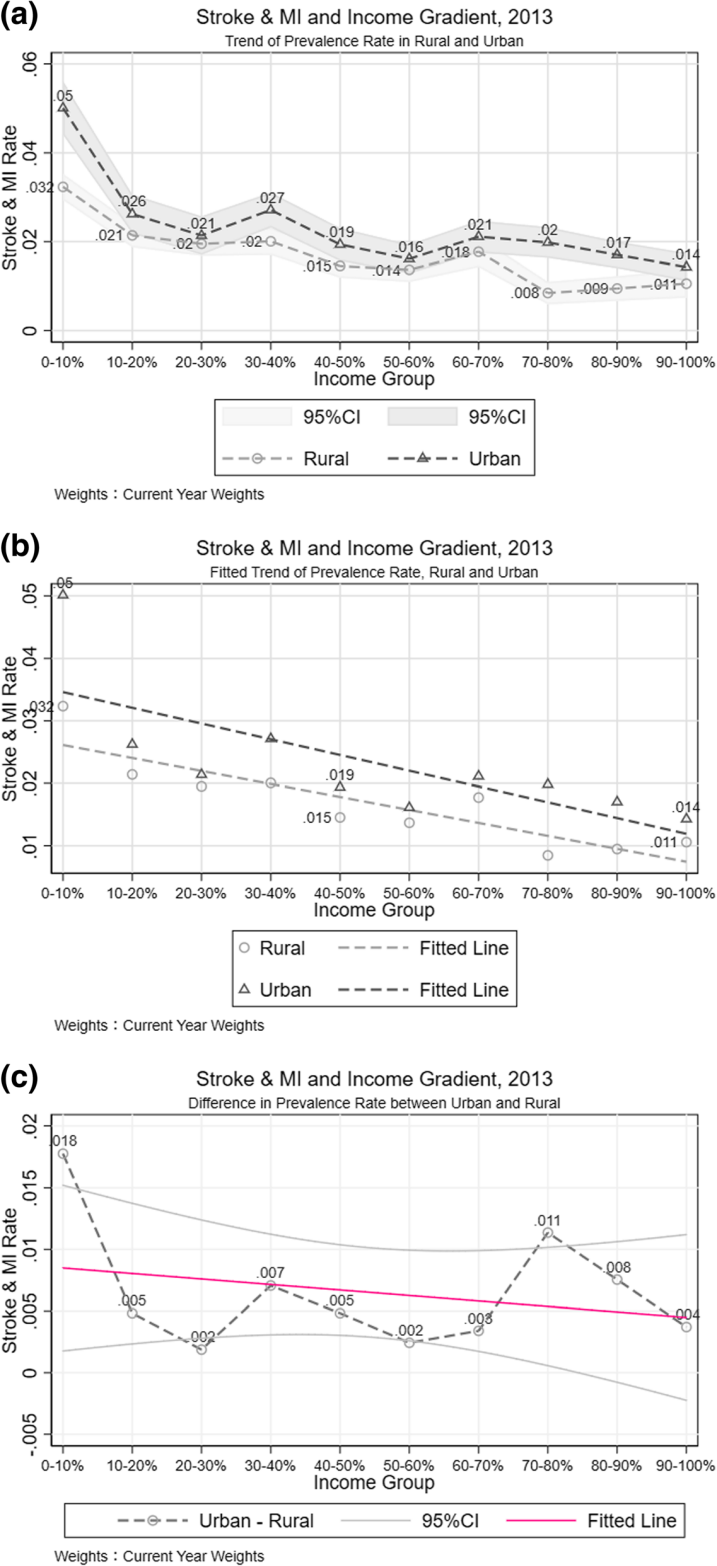
Stroke and MI Difference between Urban and Rural places. **a** Trend of Prevalence Rate in Rural and Urban. **b** Fitted Trend of Prevalence Rate, Rural and Urban. **c** Difference in Prevalence Rate between Urban and Rural

The changes in the trend of stroke and MI morbidity by income for urban and rural areas over survey waves were very different. Specifically, for rural area, the income and stroke and MI prevalence rate correlation trend had a sharper, downwardly sloped line in 2007 (Additional file 2: Figure S3-a), this phenomenon does not hold, however, in 2010 (Additional file 2: Figure S3-b) and 2013 (Fig. 4-b). The inequality in stroke and MI between rich and poor became less severe in 2013, although there was a significant increase in prevalence rate. For urban areas (Additional file 2: Figure S3 and Fig. 4), from the fitted line, income is positively correlated with reduced stroke and MI rates since 2007 as there was a slight increase in the slope of disease-income trend line.

By looking specifically into the urban-rural difference in 2013 (Fig. 4-c), conditional on the income groups divided in the full sample, urban stroke and MI prevalence rate was higher than rural prevalence rate. But most of these differences were not statistically significant, given the overlapping confidence interval of these morbidity rate. In fact, the trend of urban-rural disparity in prevalence along with income growth experienced a transition from 2007 to 2013. In 2007, along with the rise in household income, the urban-rural disparity in stroke and MI prevalence rate increased as well. In 2013, the correlation of income and urban-rural prevalence rate disparity became negative, which means, for the poorest people, the difference in stroke and MI prevalence was larger than that in the rich cohort. This negative correlation is mainly due to the huge gap in the first income decile. Table 1 Column [4, 5] provide the regression results in the urban and rural samples separately. After controlling for gender, geographic factors, job type, education background, and health behavior, relative to the first quintile income group, people who lived in rural areas with a higher income had a decline in the odds ratio of getting stroke and MI, however this effect was not statistically significant in urban areas.

### Regional difference of stroke and MI by household income

**Fig. 5:**
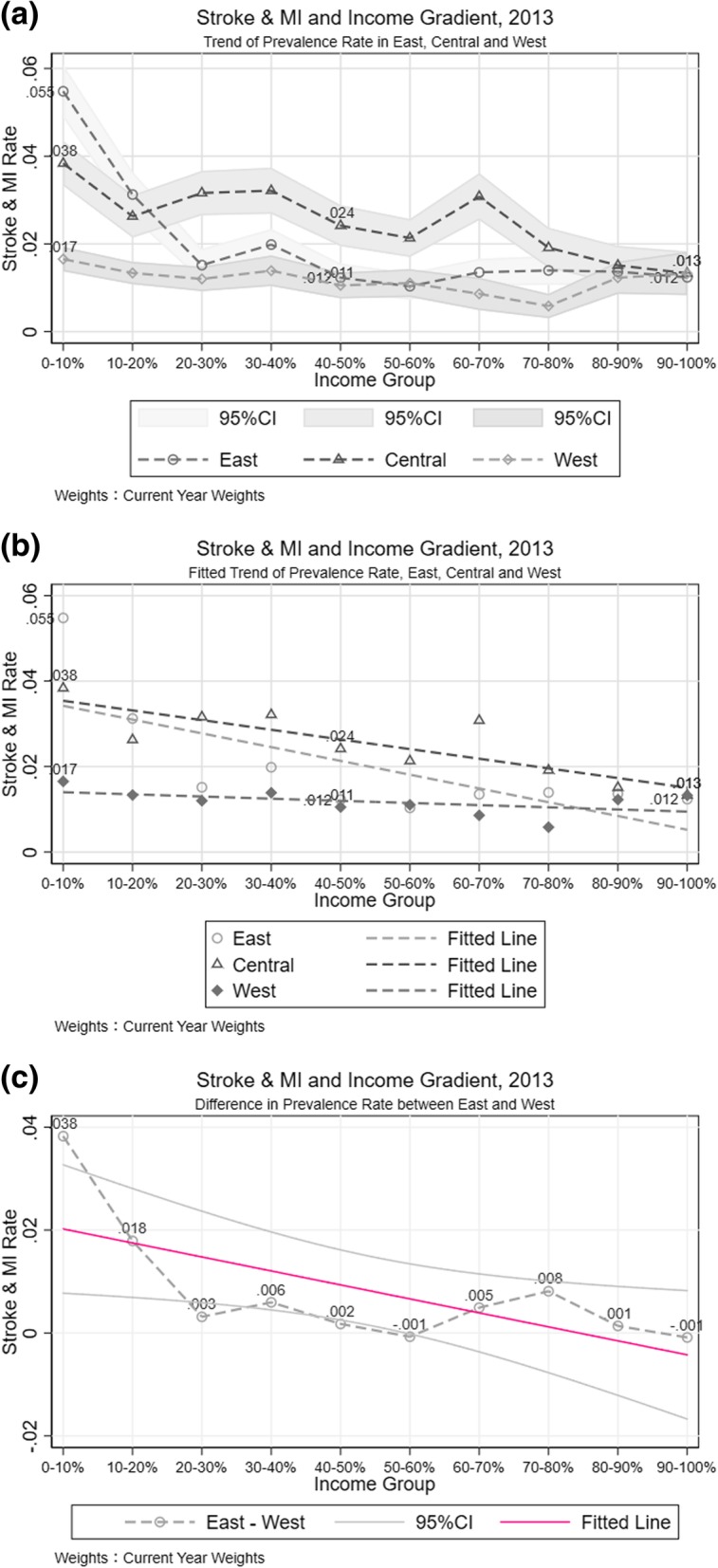
Stroke and MI Difference among Eastern, Western, and Central Regions. **a** Trend of Prevalence Rate in East, Central and West. **b** Fitted Trend of Prevalence Rate, East, Central and West. **c** Difference in Prevalence Rate between East and West

The prevalence rates in eastern, western, and central China are quite similar in 2007, but the gaps among regions enlarged over time; primarily reflected by the gap between central provinces and the rest of China. In 2013, central China possessed the highest stroke and MI rate of all three regions in upper-, middle-, and low- income decile. The morbidity rates in the three regions in the highest income decile somehow convergence (Fig. 5).

Comparing stroke and MI morbidity among the three regions of China in each survey wave showed that, in 2007, within central China, the morbidity-income correlation had a sharper rate than the other two regions (Additional file 2: Figure S4-a). This means that higher income had a similar association with stroke and MI rate for both eastern and western China, and this association was more modest than that in central China. In 2010, the gap between the eastern and western regions emerged as well (Additional file 2: Figure S4-b). Income showed its power in prohibiting the prevalence of stroke and MI in eastern and central China in both 2010 and 2013 (Fig. 5-b). This relationship was insignificant in western China, however, which means, not considering other confounding factors, the inequality in stroke and MI between rich and poor was the most moderate in western China. Those findings suggested that there may be factors other than income that affect stroke and MI prevalence among western populations. Regional morbidity inequality analysis also suggests that, moving up the income deciles, the gaps in stroke and MI risk among regions declined.

Logistic results showed that, for the whole sample consisting of three waves of survey data, relative to the bottom quintile income population, higher income levels in the eastern were connected with reductions in stroke and MI prevalence, and this effect was consistent with 2013 data analysis. The most interesting results come from the analysis of the central region. This model shows that, after controlling for age, gender, urban-rural status, education, and health behavior, the most significant decline in stroke and MI rate was the highest quintile in pooled data. The association of income with stroke and MI morbidity was quite ambiguous in 2013, which is inconsistent with as what had been shown in Fig. 5-b. Conversely, the result from the western remained consistent with Fig. 5-b. As a robustness check, we treated categorical income variables as continuous and this also yielded similar results as Table 2.

**Table 2:** Stroke and MI and Its Correlation with Income, Subsample Analysis

	Eastern	Central	Western	Eastern	Central	Western
Variables	[1]	[2]	[3]	[4]	[5]	[6]
Income						
20~40%	− 0.231**	− 0.0370	− 0.349**	− 0.115	−0.178	− 0.231**
	(− 2.097)	(− 0.358)	(− 2.026)	(− 0.793)	(− 1.017)	(− 2.097)
40~60%	− 0.504***	−0.253**	− 0.838***	−0.438***	− 0.0904	−0.504***
	(− 2.827)	(− 1.963)	(− 3.469)	(− 2.592)	(− 0.375)	(− 2.827)
60~80%	− 0.238	0.119	− 0.608**	−0.309**	− 0.183	−0.238
	(− 1.374)	(0.706)	(− 2.388)	(− 2.065)	(− 0.888)	(− 1.374)
80~100%	− 0.541***	− 0.165	− 0.971***	−0.573***	− 0.304	−0.541***
	(− 2.739)	(− 1.021)	(− 3.584)	(− 3.922)	(− 0.884)	(− 2.739)
Weighted Obs.	1,601,683,722	878,822,001	722,529,940	1,176,985,245	424,698,477	1,601,683,722
Year	Y	Y	Y	Y	Y	Y
Fixed Effects	Province	Province	Province	Province	Province	Province

Notes: Robust z-statistics in parentheses. *** *p* < 0.01, ** *p* < 0.05, * *p* < 0.1. Column [1] to [3] were regression results from three-year pooled data, Column [4] to [6] were results by using data in 2013 only. All regressions were controlled for Gender, Rural-Urban status, East, West, and Central regions, Drinking Behavior, Job Type, and Age Groups. Logistic regression results are expressed in the form of natural logarithm odds ratio

### Health behavioral difference of stroke and MI by household income

In 2007, for the poorest people in our dataset moving up one decile the stroke and MI rate fell to less than half of the prevalence in lowest income deciles (Additional file 2: Figure S5-a). Beginning with the third income decile, the stroke and MI prevalence rates of non-smokers^9^ stayed at a very low level, while that of people who had ever smoked rose a little bit moving up income deciles. From the fitted line generated through an OLS regression, the negative association of income and stroke and MI rate was stronger among non-smokers.

**Fig. 6:**
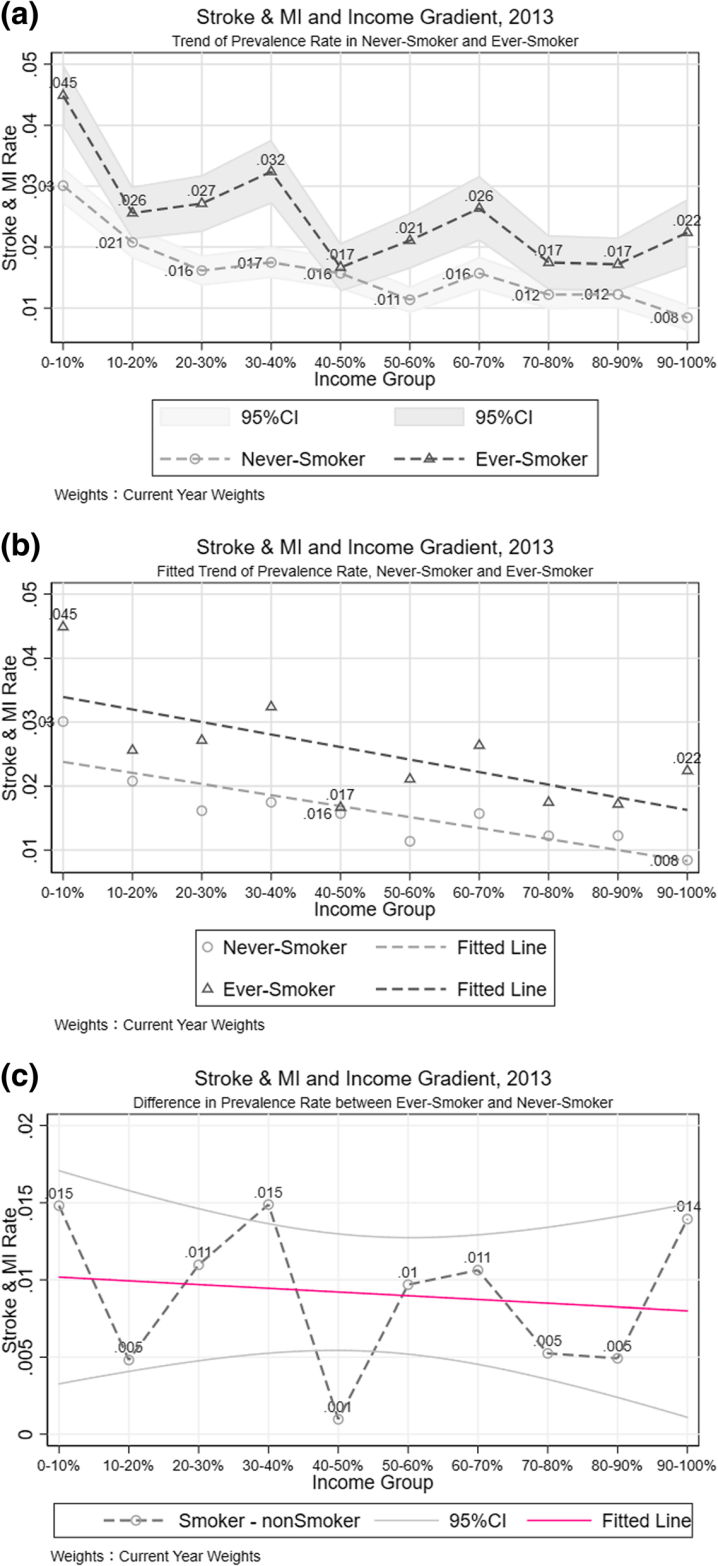
Stroke and MI Difference between Smoking Behaviors. **a** Trend of Prevalence Rate in Never-Smoker and Ever-Smoker. **b** Fitted Trend of Prevalence Rate, Never-Smoker and Ever-Smoker. **c** Difference in Prevalence Rate between Never-Smoker and Ever-Smoker

However, the relationship between income and stroke and MI prevalence in the ever-smoker population was stronger in 2010 (Additional file 2: Figure S5-b) compared with that in 2007. This made the fitted lines in both non-smoker and ever-smoker populations almost parallel. The fitted lines in Fig. 6-b also show the decreasing trend of morbidity across income gradient was consistent between ever-smoker and non-smoker, the descending trend in odds ratio with the increase in income is confirmed by empirical analysis (Table 3 Column [1, 2]).

**Table 3:** Stroke and MI and Its Correlation with Income, Subsample Analysis

	non-Smoker	ever-Smoker	Excess. Drinker	Seld. Drinker	Non-Drinker
Variables	[1]	[2]	[3]	[4]	[5]
Income					
20~40%	−0.197	− 0.00973	0.0436	− 0.497	−0.186**
	(−1.273)	(−0.125)	− 0.145	(− 1.246)	(− 2.070)
40~60%	− 0.368***	− 0.241***	−0.163	− 0.584	−0.532***
	(− 2.919)	(−2.727)	(− 0.762)	(−1.513)	(− 3.514)
60~80%	−0.0503	− 0.120	0.122	− 0.183	− 0.311**
	(− 0.249)	(− 0.827)	−0.323	(− 0.389)	(−2.013)
80~100%	−0.467*	− 0.300***	0.0774	− 0.272	−0.761***
	(− 1.949)	(−2.669)	− 0.222	(− 0.690)	(−3.736)
Weighted Obs.	1,002,012,235	592,515,561	447,570,308	341,024,681	942,302,085
Year	Y	Y	Y	Y	Y
Fixed Effects	Province	Province	Province	Province	Province

Notes: Robust z-statistics in parentheses. *** *p* < 0.01, ** *p* < 0.05, * *p* < 0.1. Regression results were from three-year pooled data. All regressions were controlled for Gender, Rural-Urban status, East, West, and Central regions, Drinking Behavior, Job Type, and Age Groups. Logistic regression results are expressed in the form of natural logarithm odds ratio

All in all, people with a smoking history ended up in a higher stroke and MI risk, (Fig. 6-b), as expected, and the gap between ever-smoker and non-smoker populations increased over time. Ever-smokers faced greater fluctuations in stroke and MI risk across income deciles looking across each income group and year. Also, there was a slight negative correlation between income and disease disparity between ever-smoker and non-smoker.

**Fig. 7:**
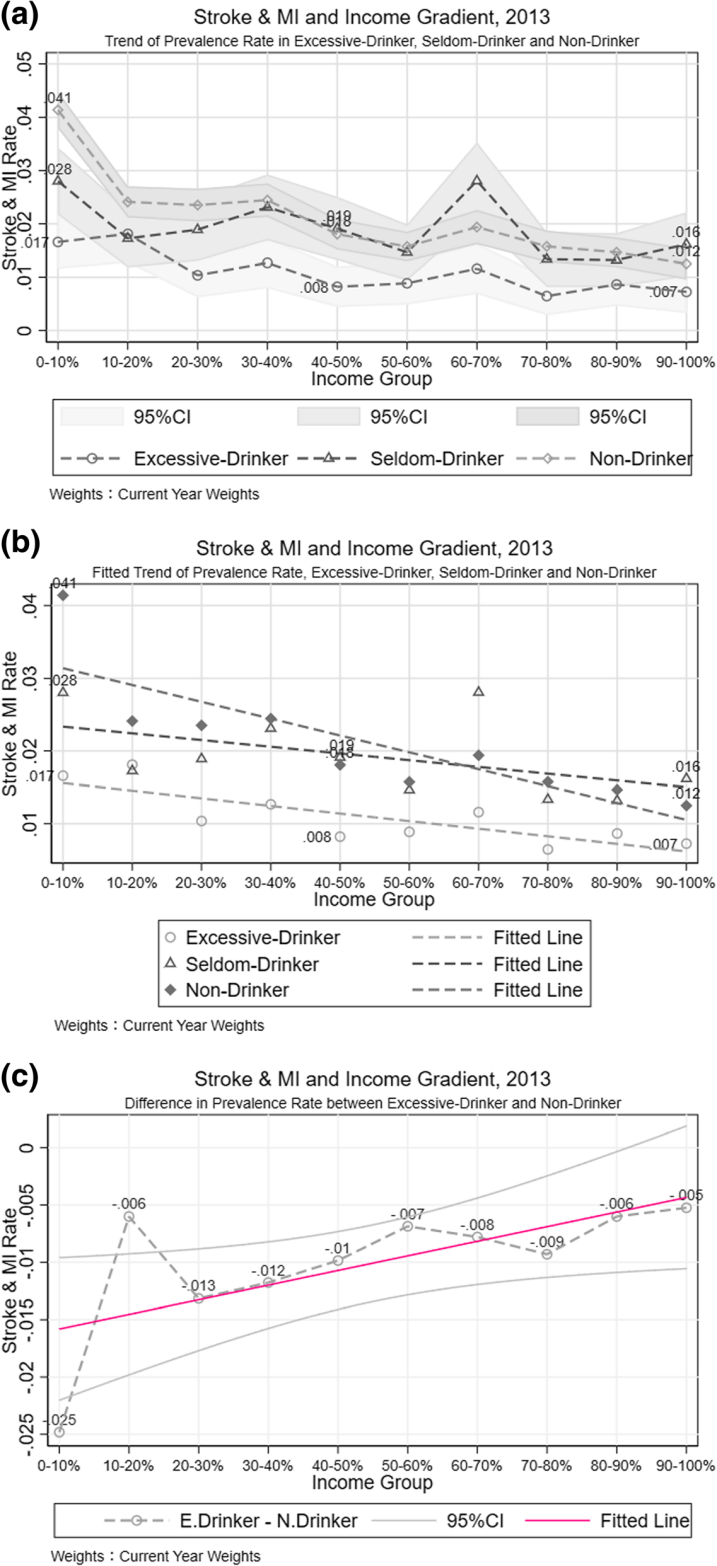
Stroke and MI Difference between Drinking Behaviors. **a** Trend of Prevalence Rate in Excessive-Drinker, Seldom-Drinker and Non-Drinker. **b** Fitted Trend of Prevalence Rate, Excessive-Drinker, Seldom-Drinker and Non-Drinker. **c** Difference in Prevalence Rate between Excessive-Drinker and Non-Drinker

By categorizing people into different drinking behavior groups, people who drink excessively in the 2010 and 2013 samples reported a lower stroke and MI risk, this relationship may reflect the self-selection in drinking behavior. The stroke and MI risk of people who seldom drank or did not drink at all had a steady, negative response to income. Additional file 2: Figure S6 shows the upward trend of the fitted line generated by the stroke and MI rate and income of drink excessively group, which indicates that a higher income was associated with a greater stroke and MI risk. Nevertheless, this trend changed in 2013 (Fig. 7-b), which once again showed that lower income was correlated with higher stroke and MI risk. The empirical analysis also showed the ambiguity in the income-morbidity linkage in population with drinking behavior (Table 3 Column [3, 5]).

## Discussion

The health and wealth linkage is often debated because the underlying policy implications play a vital role in social welfare. Therefore, identifying the health-wealth distribution built on real world data is still the essential first step in understanding this well-known connection. At the current ex-post stage of epidemiology transition, in order to detect the potential health disparity within the Chinese population and its components, such as variation in gender and urban-rural status, through implementing a national representative survey data conducted by China CDC Chronic Disease Control Center, this study explored the development of stroke and MI prevalence in each household income decile. The prevalence rate is age adjusted by using China census population in 2010.

### Key results and interpretation

#### Stroke and MI burden and its association with income

One of the main conclusions is that, from the three waves of data, the stroke and MI burden has grown significantly as stroke and MI risk reported in every income decile in 2013 almost doubled levels seen in 2010 and 2007. Looking across income deciles, there was a clear inverse relationship between income and stroke and MI morbidity. That was to say, stroke and MI risk declined in higher income deciles. The majority of this reduction occurs when moving from the lowest income decile towards higher deciles. The difference in prevalence rate between cohorts in the top 10% and bottom 10% of full sample distribution in 2013 was 1.9%.

The disease-income relationship weakened over time. On one hand, this result shows greater equality between rich and poor people—both rich and poor people face a similar stroke and MI risk. On the other hand, it is an indication that income might not be as important as it used to be in stroke and MI control and prevention, all else equal. As such, exploring the non-economic impact factors in high or low stroke and MI prevalence are even more important.

#### Stroke and MI disparity and its association with income

Prevalence rate variations across genders and geographic areas were identified. Generally, men had a higher stroke and MI rate than women. Higher income levels were linked to a decrease in stroke and MI prevalence for both sexes: the correlations for both genders were approximately parallel in 2013, even though the relationship used to be stronger in female population. Subtracting the morbidity of females from males yielded the gender gap, which decreased at higher income levels in 2013. Once again, the strength of this linkage faded over time which meant that income used to matter more. Urban populations faced a more severe stroke and MI prevalence than rural populations, and central China also faced a higher rate of stroke and MI than eastern and western China in 2010 and 2013. In addition, western China had the steadiest stroke and MI rate among those three regions. However, unlike the gap between genders, the urban-rural gap and the regional gap in stroke and MI prevalence declined at higher income levels.

#### Stroke and MI difference between health behaviors and its association with income

People who had ever smoked faced a higher risk in getting stroke and MI relative to non-smokers. However, people who drank excessively did not have higher morbidity, this may due to the endogeneity problem (self-selection) of drinking behavior. Conditional on drinking and smoking behavior, income had a negative correlation with stroke and MI rate, but the strength of this correlation changed over the years of the survey. The gap between ever-smokers and non-smokers or excessive-drinkers andnon-drinkers negatively correlated with income decile, but this correlation diminished over time.

### Limitation

There are many avenues for future work based on the findings from this study. First, in regard to the large fluctuations in morbidity analysis in some subsample analysis, the sample size should be expanded in subsequent research in order to capture a more precise stroke and MI prevalence in some characteristic-specific populations, such as cohorts with relatively low education or different occupation types, which will enable researchers to better understand time trends and changes in disadvantaged groups. Second, the correlation between stroke and MI prevalence and health care utilization should be included in the analysis, which could provide useful information on the determinants of stroke and MI prevalence in each cohort yielding better implications for policy making. Last but not least, in order to conduct a more comprehensive analysis between income and stroke and MI prevalence rate, a more detailed and comprehensive income investigation is needed in future studies.

### Comparison with other studies

The present study provided an overview of stroke and MI prevalence disparity in terms of income gradient in China, and found that, in general, richer people faced a better health condition which was quite consistent with the study conducted by Chetty et al. [7]. In which, Chetty et al. looked into the life expectancy of US people across income distribution. Another most related research came from Wang et al. [13]. Wang et al. studied the prevalence of stroke in China by using a national representative sample in 2013, and found that man had a higher stroke & MI prevalence rate than women which was also confirmed by our study. Yu et al. studied the association between social economics status and cardiovascular risk factors in Tianjin, China [14]. However, their results revealed the importance of education in enhancing population health rather than income, and education mattered more in women than men.

## Conclusion

In our national representative study, the increase in income was associated with a decrease in stroke and MI prevalence rate, which reveals the health inequality between rich and poor individuals. However, this health disparity mitigated over time. In 2013, the prevalence disparity in different income groups were similar in both sexes, and the prevalence rate of stroke and MI for male population is higher. In addition, regional heterogeneity in income associated disease disparity shows that, moving up the income deciles, the gaps in stroke and MI risk among regions narrowed down.

## Additional files

Additional file 1: Income and prevalence rate of Stroke and MI, statistics and correlations. (DOCX 41 kb)

Additional file 2: Supplementary figures of the prevalence rate of Storke and MI across income groups in subgroups. (PDF 487 kb)

## Data Availability

The datasets generated and/or analysed during the current study are not publicly available due confidentiality agreement but are available from the corresponding author on reasonable request.

## Abbreviations

Myocardial infarction (MI): commonly known as a heart attack, occurs when blood flow decreases or stops to a part of the heart, causing damage to the heart muscle. The most common symptom is chest pain or discomfort which may travel into the shoulder, arm, back, neck, or jaw.

## Footnotes

1 https://data.worldbank.org/indicator/NY.GDP.MKTP.PP.KD, Based on PPP estimates.

2 WHO Mortality Database. http://apps.who.int/healthinfo/statistics/mortality/whodpms/

3
http://www.healthdata.org/data-visualization/gbd-compare

4 The weighting factor is applied to the sampling scheme with inconsistent sample selection probabilities, and the post-adjustment weights, which makes the sample structure of the survey consistent with the sample structure of the 2010 China Census.

5 The missing of ID and Weights means that the record is incomplete or incorrect in raw data. Sample characteristics in both missing-data-group and non-missing-data group is compared and available under request.

6 The data for 2007 is based on the diagnosis of doctors in primary health care institutions; in 2010, it was based on the presence of disease attack. Therefore, in 2010, some people were diagnosed but did not experience a disease attack within past 12 months, some may experience a disease attack without being diagnosed. So, compared with 2007, the measured prevalence of stroke and MI in 2010 is biased. The stroke and MI relevant question in 2013 is similar to that in 2007 except for the type of medical institutions included in. Since some respondents are not diagnosed by the county-level medical institutions or above, so the measured prevalence rate may be underestimated. At the same time, the costs of different medical institutions are different, so those who do not go to the medical institutions at or above the county level may because they cannot afford. Thus, compared with 2007, the 2013 question may lead to an underestimation of prevalence rate within low-income population.

7 Due to the limited questions in questionnaires, we define the ever-smoker and non-smoker from the following questions: 1. Do you smoke currently? 2. Have you ever smoked?

8 In addition, the correlation analysis between disease prevalence and income was also computed, and all turned out to be insignificant.

9 People is categorized into ever smoked group and never smoked group due to data according to questionnaire.
