# Supplementary material for: Disease and disparity in China: a view from stroke and MI disease
Source: Int J Equity Health. 2019 Jun 11;18:85. doi: 10.1186/s12939-019-0986-2 (PMC6558805; doi:10.1186/s12939-019-0986-2)
Supplement: Supplementary file 1 — Income and prevalence rate of Stroke and MI, statistics and correlations. (DOCX 41 kb) [file 12939_2019_986_MOESM1_ESM.docx]

**Appendix**

Tables

**Table A1-1 Average Income in Each Wave of Survey**

| **Inc. Group** | **2007** | | | **2010** | | | **2013** | | |
| --- | --- | --- | --- | --- | --- | --- | --- | --- | --- |
|  | **Mean** | **Obs.** | **Weighted Obs.** | **Mean** | **Obs.** | **Weighted Obs.** | **Mean** | **Obs.** | **Weighted Obs.** |
| **0~10%** | 2,433.331 | 5,067 | 77,036,456 | 3,257.466 | 8,223 | 77,895,184 | 5,701.608 | 17,878 | 74,075,256 |
| **10-20%** | 5,303.716 | 4,430 | 76,973,648 | 7,452.542 | 8,158 | 77,884,624 | 13,504.460 | 15,141 | 74,075,464 |
| **20~30%** | 7,839.745 | 3,997 | 77,104,672 | 9,835.521 | 7,660 | 77,917,216 | 18,380.870 | 13,672 | 74,069,216 |
| **30~40%** | 9,960.315 | 3,966 | 77,026,736 | 13,325.940 | 8,838 | 77,896,248 | 24,158.500 | 13,665 | 74,080,528 |
| **40~50%** | 11,820.120 | 4,416 | 77,049,080 | 17,837.880 | 7,917 | 77,900,160 | 28,455.150 | 12,339 | 74,077,440 |
| **50~60%** | 15,973.410 | 4,384 | 77,037,248 | 20,838.820 | 7,806 | 77,893,808 | 35,567.010 | 11,899 | 74,074,000 |
| **60~70%** | 20,327.910 | 4,075 | 77,026,240 | 26,602.940 | 7,791 | 77,885,208 | 41,740.300 | 11,076 | 73,683,712 |
| **70~80%** | 25,745.580 | 4,129 | 77,045,632 | 32,703.540 | 7,816 | 77,916,864 | 50,570.150 | 10,614 | 74,468,944 |
| **80~90%** | 35,088.230 | 4,050 | 77,026,008 | 43,980.010 | 7,455 | 77,895,608 | 65,638.600 | 11,214 | 74,039,008 |
| **90~100%** | 89,291.410 | 3,928 | 77,058,240 | 90,521.920 | 7,222 | 77,905,608 | 134,916.400 | 9,010 | 74,112,440 |

Notes: In 2007, the time frame of income question is “over the past 12 months”. The 2010 income time frame is “in 2009”. The 2013 income time frame is “in 2012”. Therefore, the time frame of 2010 and 2013 survey is consistent while the 2007 is less comparable. The household income surveyed is not equivalized by using any household equivalence scale. Income is adjusted to US dollars based on the exchange rate of corresponding year.

**Table A1-2 Prevalence Rate of Stroke & MI in Each Wave of Survey**

| **Inc. Group** | **2007** | | | **2010** | | | **2013** | | |  |
| --- | --- | --- | --- | --- | --- | --- | --- | --- | --- | --- |
|  | **Prevalence Rate(%)** | **95% CI** | | **Prevalence Rate(%)** | **95% CI** | | **Prevalence Rate(%)** | **95% CI** | |  |
| **0~10%** | 3.290 | 2.804 | 3.777 | 1.238 | 1.002 | 1.473 | 3.021 | 2.771 | 3.271 | |
| **10-20%** | 1.869 | 1.482 | 2.256 | 0.988 | 0.784 | 1.192 | 1.799 | 1.588 | 2.009 | |
| **20~30%** | 1.289 | 0.963 | 1.614 | 0.968 | 0.758 | 1.178 | 1.980 | 1.756 | 2.204 | |
| **30~40%** | 0.927 | 0.623 | 1.232 | 0.747 | 0.560 | 0.935 | 2.281 | 2.035 | 2.527 | |
| **40~50%** | 1.009 | 0.733 | 1.285 | 0.742 | 0.551 | 0.934 | 1.718 | 1.504 | 1.931 | |
| **50~60%** | 0.961 | 0.674 | 1.248 | 0.498 | 0.342 | 0.655 | 1.671 | 1.455 | 1.888 | |
| **60~70%** | 0.707 | 0.453 | 0.960 | 0.634 | 0.451 | 0.818 | 2.111 | 1.866 | 2.355 | |
| **70~80%** | 0.789 | 0.517 | 1.062 | 0.625 | 0.453 | 0.797 | 1.869 | 1.627 | 2.111 | |
| **80~90%** | 1.105 | 0.785 | 1.425 | 0.553 | 0.377 | 0.728 | 1.886 | 1.633 | 2.139 | |
| **90~100%** | 0.800 | 0.515 | 1.085 | 0.430 | 0.272 | 0.588 | 1.435 | 1.201 | 1.669 | |

**Table A1-3 Subsample Size in Each Wave of Survey**

| **Subsample** | **2007** | | **2010** | | **2013** | |
| --- | --- | --- | --- | --- | --- | --- |
|  | **Obs.** | **Weighted Obs.** | **Obs.** | **Weighted Obs.** | **Obs.** | **Weighted Obs.** |
| **Male** | 20,089 | 393,721,632 | 36,511 | 399,851,008 | 55,193 | 377,277,248 |
| **Female** | 22,353 | 376,662,336 | 42,375 | 379,139,520 | 71,315 | 363,478,752 |
| **East** | 14,251 | 304,946,944 | 25,126 | 301,711,680 | 46,691 | 303,859,712 |
| **Central** | 13,181 | 250,872,560 | 24,971 | 255,434,704 | 36,992 | 240,800,448 |
| **West** | 15,010 | 214,564,432 | 28,789 | 221,844,144 | 42,825 | 196,095,824 |
| **Rural** | 26,014 | 526,524,064 | 48,587 | 542,023,168 | 72,879 | 475,452,544 |
| **Urban** | 16,428 | 243,859,888 | 30,299 | 236,967,328 | 53,629 | 265,303,440 |
| **Age 18~44** | 20,816 | 468,376,448 | 34,036 | 429,939,616 | 39,184 | 407,873,088 |
| **Age 45~59** | 14,348 | 206,305,792 | 27,071 | 207,668,000 | 49,720 | 193,096,816 |
| **Age 60+** | 5,839 | 71,662,632 | 15,697 | 122,022,392 | 34,264 | 119,346,224 |
| **Ever-Smoker** | 27,441 | 488,497,568 | 52,991 | 511,240,672 | 88,008 | 498,786,976 |
| **Never-Smoker** | 14,762 | 274,408,896 | 25,774 | 266,627,024 | 38,430 | 241,744,528 |
| **Excessive-Drinker** | 5,353 | 92,159,568 | 15,917 | 164,478,880 | 21,420 | 149,032,304 |
| **Seldom-Drinker** | 10,362 | 208,950,992 | 12,238 | 126,463,352 | 20,826 | 131,076,672 |
| **Non-Drinker** | 26,696 | 468,811,648 | 50,726 | 487,994,528 | 84,255 | 460,624,864 |

**Table A2 the Distribution of Income**

| **Year** | **Inc Group** | **Without Stroke & MI** | | **With Stroke or MI** | | **T** |
| --- | --- | --- | --- | --- | --- | --- |
|  |  | **Average Income** | **Obs.** | **Average Income** | **Obs.** |  |
| **2007** | 0~10% | 299.2265 | 4,567 | 283.8433 | 143 | 1.4192 |
|  | 10%~20% | 673.8202 | 4,251 | 676.1972 | 66 | -0.2028 |
|  | 20%~30% | 970.6073 | 3,828 | 979.5328 | 53 | -0.5911 |
|  | 30%~40% | 1302.75 | 3,827 | 1293.664 | 61 | 0.7459 |
|  | 40%~50% | 1543.586 | 4,236 | 1560.192 | 66 | -1.0772 |
|  | 50%~60% | 2049.854 | 4,223 | 2087.032 | 45 | -1.2121 |
|  | 60%~70% | 2684.936 | 3,947 | 2683.01 | 52 | 0.0949 |
|  | 70%~80% | 3465.737 | 4,003 | 3449.324 | 57 | 0.3938 |
|  | 80%~90% | 4772.469 | 3,928 | 4842.82 | 60 | -1.1091 |
|  | 90%~100% | 12026.68 | 3,835 | 10284.53 | 49 | 0.3588 |
| **2010** | 0~10% | 372.4091 | 8,054 | 366.1416 | 153 | 0.4663 |
|  | 10%~20% | 830.3012 | 8,029 | 825.377 | 120 | 0.3833 |
|  | 20%~30% | 1285.577 | 7,564 | 1289.435 | 88 | -0.368 |
|  | 30%~40% | 1611.109 | 8,718 | 1619.04 | 110 | -0.4378 |
|  | 40%~50% | 2318.672 | 7,831 | 2364.836 | 86 | -1.693 |
|  | 50%~60% | 2848.544 | 7,720 | 2902.218 | 81 | -2.1685 |
|  | 60%~70% | 3602.234 | 7,694 | 3597.639 | 89 | 0.1187 |
|  | 70%~80% | 4546.083 | 7,726 | 4515.265 | 87 | 0.7811 |
|  | 80%~90% | 6082.049 | 7,387 | 6041.456 | 66 | 0.5667 |
|  | 90%~100% | 12618.25 | 7,158 | 11310.62 | 57 | 0.3581 |
| **2013** | 0~10% | 769.6222 | 17,097 | 741.3148 | 740 | 2.0117 |
|  | 10%~20% | 1659.962 | 14,662 | 1735.219 | 455 | -5.0278 |
|  | 20%~30% | 2603.825 | 13,269 | 2607.174 | 372 | -0.5976 |
|  | 30%~40% | 3424.035 | 13,190 | 3411.775 | 443 | 0.7276 |
|  | 40%~50% | 4191.417 | 12,011 | 4321.634 | 305 | -6.6983 |
|  | 50%~60% | 5043.729 | 11,626 | 5030.299 | 253 | 0.7537 |
|  | 60%~70% | 6302.003 | 10,749 | 6247.902 | 303 | 3.2705 |
|  | 70%~80% | 7545.894 | 10,347 | 7600.957 | 243 | -1.6506 |
|  | 80%~90% | 9653.946 | 10,905 | 9636.09 | 270 | 0.2806 |
|  | 90%~100% | 20091.27 | 8,760 | 20506.59 | 218 | -0.2919 |

Notes: Income is in US dollars. For most of the income groups, the difference in income is not significant between cohort with stoke & MI and cohort without stroke & MI.

**Table A3-1 the Correlation between Income and Prevalence Rate across Income Groups, Categorical Income, 2007**

|  | (1) | (2) | (3) | (4) | (5) | (6) | (7) |
| --- | --- | --- | --- | --- | --- | --- | --- |
|  | Prevalence Rate in | Prevalence Rate in | Prevalence Rate in | Prevalence Rate in | Prevalence Rate in | Prevalence Rate in | Prevalence Rate in |
| VARIABLES | Female | Male | Rural | Urban | Age 18~44 | Age 45~59 | Age 60+ |
| Inc Group | -0.00298*** | -0.000467 | -0.00253*** | -0.000802 | -0.00159** | -0.000687 | -0.000945 |
|  | (-3.733) | (-0.856) | (-4.107) | (-1.063) | (-2.375) | (-0.950) | (-0.735) |
| Constant | 0.0301*** | 0.0142*** | 0.0242*** | 0.0198*** | 0.0140*** | 0.0218*** | 0.0554*** |
|  | (6.069) | (4.202) | (6.321) | (4.223) | (3.379) | (4.862) | (6.940) |
| Observations | 10 | 10 | 10 | 10 | 10 | 10 | 10 |
| R-squared | 0.635 | 0.084 | 0.678 | 0.124 | 0.413 | 0.101 | 0.063 |

Notes: T-statistics in parentheses. *** p<0.01, ** p<0.05, * p<0.1

**Table A3-2 the Correlation between Income and Prevalence Rate across Income Groups, Categorical Income, 2007**

|  | (1) | (2) | (3) | (4) | (5) | (6) | (7) | (8) |
| --- | --- | --- | --- | --- | --- | --- | --- | --- |
|  | Prevalence Rate in | Prevalence Rate in | Prevalence Rate in | Prevalence Rate in | Prevalence Rate in | Prevalence Rate in | Prevalence Rate in | Prevalence Rate in |
| VARIABLES | East | Central | West | Ever-Smoker | Never-Smoker | Excessive Drinker | Seldom-Drinker | Non-Drinker |
| Inc Group | -0.00133* | -0.00249** | -0.00124 | -0.00154** | -0.000242 | 0.000185 | -0.000650 | -0.00239** |
|  | (-2.096) | (-2.688) | (-1.395) | (-2.630) | (-0.521) | (0.350) | (-0.839) | (-2.630) |
| Constant | 0.0194*** | 0.0278*** | 0.0192*** | 0.0187*** | 0.0149*** | 0.00829** | 0.0128** | 0.0278*** |
|  | (4.906) | (4.825) | (3.479) | (5.133) | (5.155) | (2.535) | (2.671) | (4.921) |
| Observations | 10 | 10 | 10 | 10 | 10 | 10 | 10 | 10 |
| R-squared | 0.355 | 0.474 | 0.196 | 0.464 | 0.033 | 0.015 | 0.081 | 0.464 |

Notes: T-statistics in parentheses. *** p<0.01, ** p<0.05, * p<0.1

**Table A4-1 the Correlation between Income and Prevalence Rate across Income Groups, Categorical Income, 2010**

|  | (1) | (2) | (3) | (4) | (5) | (6) | (7) |
| --- | --- | --- | --- | --- | --- | --- | --- |
|  | Prevalence Rate in | Prevalence Rate in | Prevalence Rate in | Prevalence Rate in | Prevalence Rate in | Prevalence Rate in | Prevalence Rate in |
| VARIABLES | Female | Male | Rural | Urban | Age 18~44 | Age 45~59 | Age 60+ |
| Inc Group | -0.00109*** | -0.000618*** | -0.000900*** | -0.00174*** | -1.20e-05 | -0.00111*** | 0.000865 |
|  | (-4.091) | (-3.487) | (-4.889) | (-5.960) | (-0.0852) | (-3.759) | (1.560) |
| Constant | 0.0141*** | 0.0136*** | 0.0124*** | 0.0237*** | 0.00266** | 0.0177*** | 0.0259*** |
|  | (8.548) | (12.35) | (10.89) | (13.07) | (3.031) | (9.627) | (7.522) |
| Observations | 10 | 10 | 10 | 10 | 10 | 10 | 10 |
| R-squared | 0.677 | 0.603 | 0.749 | 0.816 | 0.001 | 0.638 | 0.233 |

Notes: T-statistics in parentheses. *** p<0.01, ** p<0.05, * p<0.1

**Table A4-2 the Correlation between Income and Prevalence Rate across Income Groups, Categorical Income, 2010**

|  | (1) | (2) | (3) | (4) | (5) | (6) | (7) | (8) |
| --- | --- | --- | --- | --- | --- | --- | --- | --- |
|  | Prevalence Rate in | Prevalence Rate in | Prevalence Rate in | Prevalence Rate in | Prevalence Rate in | Prevalence Rate in | Prevalence Rate in | Prevalence Rate in |
| VARIABLES | East | Central | West | Ever-Smoker | Never-Smoker | Excessive Drinker | Seldom-Drinker | Non-Drinker |
| Inc Group | -0.00103** | -0.00136*** | -0.000327 | -0.000838*** | -0.000843** | 8.07e-05 | -0.000659** | -0.00108*** |
|  | (-3.289) | (-4.691) | (-1.596) | (-3.862) | (-3.298) | (0.553) | (-2.667) | (-5.200) |
| Constant | 0.0152*** | 0.0187*** | 0.00888*** | 0.0123*** | 0.0168*** | 0.00506*** | 0.0126*** | 0.0162*** |
|  | (7.803) | (10.37) | (6.971) | (9.109) | (10.58) | (5.589) | (8.222) | (12.63) |
| Observations | 10 | 10 | 10 | 10 | 10 | 10 | 10 | 10 |
| R-squared | 0.575 | 0.733 | 0.241 | 0.651 | 0.576 | 0.037 | 0.471 | 0.772 |

Notes: T-statistics in parentheses. *** p<0.01, ** p<0.05, * p<0.1

**Table A5-1 the Correlation between Income and Prevalence Rate across Income Groups, Categorical Income, 2013**

|  | (1) | (2) | (3) | (4) | (5) | (6) | (7) |
| --- | --- | --- | --- | --- | --- | --- | --- |
|  | Prevalence Rate in | Prevalence Rate in | Prevalence Rate in | Prevalence Rate in | Prevalence Rate in | Prevalence Rate in | Prevalence Rate in |
| VARIABLES | Female | Male | Rural | Urban | Age 18~44 | Age 45~59 | Age 60+ |
| Inc Group | -0.00189*** | -0.00199*** | -0.00206*** | -0.00269*** | -0.000144 | -0.00185*** | 0.00120 |
|  | (-4.936) | (-3.523) | (-4.836) | (-3.535) | (-0.463) | (-5.968) | (1.513) |
| Constant | 0.0269*** | 0.0320*** | 0.0281*** | 0.0377*** | 0.00383* | 0.0319*** | 0.0640*** |
|  | (11.29) | (9.122) | (10.64) | (7.978) | (1.987) | (16.54) | (13.06) |
| Observations | 10 | 10 | 10 | 10 | 10 | 10 | 10 |
| R-squared | 0.753 | 0.608 | 0.745 | 0.610 | 0.026 | 0.817 | 0.223 |

Notes: T-statistics in parentheses. *** p<0.01, ** p<0.05, * p<0.1

**Table A5-2 the Correlation between Income and Prevalence Rate across Income Groups, Categorical Income, 2013**

|  | (1) | (2) | (3) | (4) | (5) | (6) | (7) | (8) |
| --- | --- | --- | --- | --- | --- | --- | --- | --- |
|  | Prevalence Rate in | Prevalence Rate in | Prevalence Rate in | Prevalence Rate in | Prevalence Rate in | Prevalence Rate in | Prevalence Rate in | Prevalence Rate in |
| VARIABLES | East | Central | West | Ever-Smoker | Never-Smoker | Excessive Drinker | Seldom-Drinker | Non-Drinker |
| Inc Group | -0.00339** | -0.00235*** | -0.000496 | -0.00177*** | -0.00215** | -0.00104*** | -0.000925 | -0.00232*** |
|  | (-3.055) | (-4.433) | (-1.577) | (-4.509) | (-2.957) | (-3.887) | (-1.659) | (-4.446) |
| Constant | 0.0381*** | 0.0380*** | 0.0143*** | 0.0255*** | 0.0370*** | 0.0166*** | 0.0243*** | 0.0337*** |
|  | (5.547) | (11.54) | (7.325) | (10.45) | (8.179) | (9.971) | (7.013) | (10.42) |
| Observations | 10 | 10 | 10 | 10 | 10 | 10 | 10 | 10 |
| R-squared | 0.538 | 0.711 | 0.237 | 0.718 | 0.522 | 0.654 | 0.256 | 0.712 |

Notes: T-statistics in parentheses. *** p<0.01, ** p<0.05, * p<0.1

**Table A6-1 the Correlation between Income and Prevalence Rate across Income Groups, Continuous Income, 2007**

|  | (1) | (2) | (3) | (4) | (5) | (6) | (7) |
| --- | --- | --- | --- | --- | --- | --- | --- |
|  | Prevalence Rate in | Prevalence Rate in | Prevalence Rate in | Prevalence Rate in | Prevalence Rate in | Prevalence Rate in | Prevalence Rate in |
| VARIABLES | Female | Male | Rural | Urban | Age 18~44 | Age 45~59 | Age 60+ |
| Average Inc | -2.45e-07 | 1.01e-09 | -2.04e-07 | -5.88e-08 | -1.02e-07 | -7.50e-08 | -1.15e-07 |
|  | (-1.655) | (0.0137) | (-1.682) | (-0.573) | (-0.943) | (-0.786) | (-0.688) |
| Constant | 0.0188*** | 0.0116*** | 0.0146*** | 0.0166*** | 0.00744* | 0.0196*** | 0.0526*** |
|  | (4.161) | (5.144) | (3.926) | (5.296) | (2.260) | (6.728) | (10.27) |
| Observations | 10 | 10 | 10 | 10 | 10 | 10 | 10 |
| R-squared | 0.255 | 0.000 | 0.261 | 0.039 | 0.100 | 0.072 | 0.056 |

Notes: T-statistics in parentheses. *** p<0.01, ** p<0.05, * p<0.1

**Table A6-2 the Correlation between Income and Prevalence Rate across Income Groups, Continuous Income, 2007**

|  | (1) | (2) | (3) | (4) | (5) | (6) | (7) | (8) |
| --- | --- | --- | --- | --- | --- | --- | --- | --- |
|  | Prevalence Rate in | Prevalence Rate in | Prevalence Rate in | Prevalence Rate in | Prevalence Rate in | Prevalence Rate in | Prevalence Rate in | Prevalence Rate in |
| VARIABLES | East | Central | West | Ever-Smoker | Never-Smoker | Excessive Drinker | Seldom-Drinker | Non-Drinker |
| Average Inc | -1.08e-07 | -2.06e-07 | 1.83e-08 | -1.20e-07 | 2.20e-08 | 3.67e-08 | -3.93e-08 | -1.75e-07 |
|  | (-1.130) | (-1.380) | (0.142) | (-1.267) | (0.362) | (0.543) | (-0.378) | (-1.178) |
| Constant | 0.0143*** | 0.0184*** | 0.0120** | 0.0127*** | 0.0131*** | 0.00853*** | 0.0101** | 0.0183*** |
|  | (4.902) | (4.033) | (3.050) | (4.390) | (7.026) | (4.121) | (3.176) | (4.026) |
| Observations | 10 | 10 | 10 | 10 | 10 | 10 | 10 | 10 |
| R-squared | 0.138 | 0.192 | 0.003 | 0.167 | 0.016 | 0.036 | 0.018 | 0.148 |

Notes: T-statistics in parentheses. *** p<0.01, ** p<0.05, * p<0.1

**Table A7-1 the Correlation between Income and Prevalence Rate across Income Groups, Continuous Income, 2010**

|  | (1) | (2) | (3) | (4) | (5) | (6) | (7) |
| --- | --- | --- | --- | --- | --- | --- | --- |
|  | Prevalence Rate in | Prevalence Rate in | Prevalence Rate in | Prevalence Rate in | Prevalence Rate in | Prevalence Rate in | Prevalence Rate in |
| VARIABLES | Female | Male | Rural | Urban | Age 18~44 | Age 45~59 | Age 60+ |
| Average Inc | -9.48e-08* | -7.52e-08** | -8.36e-08** | -1.77e-07** | -1.20e-08 | -1.06e-07* | 5.19e-08 |
|  | (-2.007) | (-3.343) | (-2.416) | (-3.131) | (-0.705) | (-2.214) | (0.681) |
| Constant | 0.0105*** | 0.0120*** | 0.00954*** | 0.0185*** | 0.00289*** | 0.0142*** | 0.0294*** |
|  | (6.546) | (15.81) | (8.154) | (9.658) | (5.024) | (8.733) | (11.39) |
| Observations | 10 | 10 | 10 | 10 | 10 | 10 | 10 |
| R-squared | 0.335 | 0.583 | 0.422 | 0.551 | 0.059 | 0.380 | 0.055 |

Notes: T-statistics in parentheses. *** p<0.01, ** p<0.05, * p<0.1

**Table A7-2 the Correlation between Income and Prevalence Rate across Income Groups, Continuous Income, 2010**

|  | (1) | (2) | (3) | (4) | (5) | (6) | (7) | (8) |
| --- | --- | --- | --- | --- | --- | --- | --- | --- |
|  | Prevalence Rate in | Prevalence Rate in | Prevalence Rate in | Prevalence Rate in | Prevalence Rate in | Prevalence Rate in | Prevalence Rate in | Prevalence Rate in |
| VARIABLES | East | Central | West | Ever-Smoker | Never-Smoker | Excessive Drinker | Seldom-Drinker | Non-Drinker |
| Average Inc | -9.96e-08* | -1.27e-07** | -4.44e-08 | -7.89e-08* | -9.43e-08** | -4.73e-09 | -6.99e-08* | -1.04e-07** |
|  | (-2.068) | (-2.398) | (-1.806) | (-2.197) | (-2.661) | (-0.258) | (-2.056) | (-2.660) |
| Constant | 0.0120*** | 0.0143*** | 0.00817*** | 0.00959*** | 0.0145*** | 0.00562*** | 0.0107*** | 0.0129*** |
|  | (7.354) | (7.980) | (9.822) | (7.903) | (12.08) | (9.065) | (9.304) | (9.728) |
| Observations | 10 | 10 | 10 | 10 | 10 | 10 | 10 | 10 |
| R-squared | 0.348 | 0.418 | 0.290 | 0.376 | 0.470 | 0.008 | 0.346 | 0.469 |

Notes: T-statistics in parentheses. *** p<0.01, ** p<0.05, * p<0.1

**Table A8-1 the Correlation between Income and Prevalence Rate across Income Groups, Continuous Income, 2010**

|  | (1) | (2) | (3) | (4) | (5) | (6) | (7) |
| --- | --- | --- | --- | --- | --- | --- | --- |
|  | Prevalence Rate in | Prevalence Rate in | Prevalence Rate in | Prevalence Rate in | Prevalence Rate in | Prevalence Rate in | Prevalence Rate in |
| VARIABLES | Female | Male | Rural | Urban | Age 18~44 | Age 45~59 | Age 60+ |
| Average Inc | -1.21e-07** | -1.32e-07* | -1.31e-07** | -1.76e-07* | -1.84e-08 | -1.36e-07*** | 1.02e-07 |
|  | (-2.482) | (-2.204) | (-2.424) | (-2.161) | (-0.716) | (-3.577) | (1.541) |
| Constant | 0.0212*** | 0.0262*** | 0.0219*** | 0.0298*** | 0.00376** | 0.0270*** | 0.0666*** |
|  | (8.372) | (8.426) | (7.831) | (7.042) | (2.821) | (13.73) | (19.39) |
| Observations | 10 | 10 | 10 | 10 | 10 | 10 | 10 |
| R-squared | 0.435 | 0.378 | 0.423 | 0.369 | 0.060 | 0.615 | 0.229 |

Notes: T-statistics in parentheses. *** p<0.01, ** p<0.05, * p<0.1

**Table A8-2 the Correlation between Income and Prevalence Rate across Income Groups, Continuous Income, 2010**

|  | (1) | (2) | (3) | (4) | (5) | (6) | (7) | (8) |
| --- | --- | --- | --- | --- | --- | --- | --- | --- |
|  | Prevalence Rate in | Prevalence Rate in | Prevalence Rate in | Prevalence Rate in | Prevalence Rate in | Prevalence Rate in | Prevalence Rate in | Prevalence Rate in |
| VARIABLES | East | Central | West | Ever-Smoker | Never-Smoker | Excessive Drinker | Seldom-Drinker | Non-Drinker |
| Average Inc | -1.94e-07 | -1.83e-07*** | -9.11e-09 | -1.22e-07** | -1.26e-07 | -6.78e-08* | -6.25e-08 | -1.53e-07** |
|  | (-1.625) | (-3.504) | (-0.302) | (-2.706) | (-1.643) | (-2.249) | (-1.254) | (-2.498) |
| Constant | 0.0271*** | 0.0322*** | 0.0119*** | 0.0205*** | 0.0300*** | 0.0135*** | 0.0216*** | 0.0269*** |
|  | (4.386) | (11.89) | (7.626) | (8.778) | (7.551) | (8.666) | (8.377) | (8.474) |
| Observations | 10 | 10 | 10 | 10 | 10 | 10 | 10 | 10 |
| R-squared | 0.248 | 0.605 | 0.011 | 0.478 | 0.252 | 0.387 | 0.164 | 0.438 |

Notes: T-statistics in parentheses. *** p<0.01, ** p<0.05, * p<0.1
