# Supplementary material for: Disease and disparity in China: a view from stroke and MI disease
Source: Int J Equity Health. 2019 Jun 11;18:85. doi: 10.1186/s12939-019-0986-2 (PMC6558805; doi:10.1186/s12939-019-0986-2)
Supplement: Supplementary file 2 — Supplementary figures of the prevalence rate of Storke and MI across income groups in subgroups. (PDF 487 kb) [file 12939_2019_986_MOESM2_ESM.pdf]

Appendix

Figures

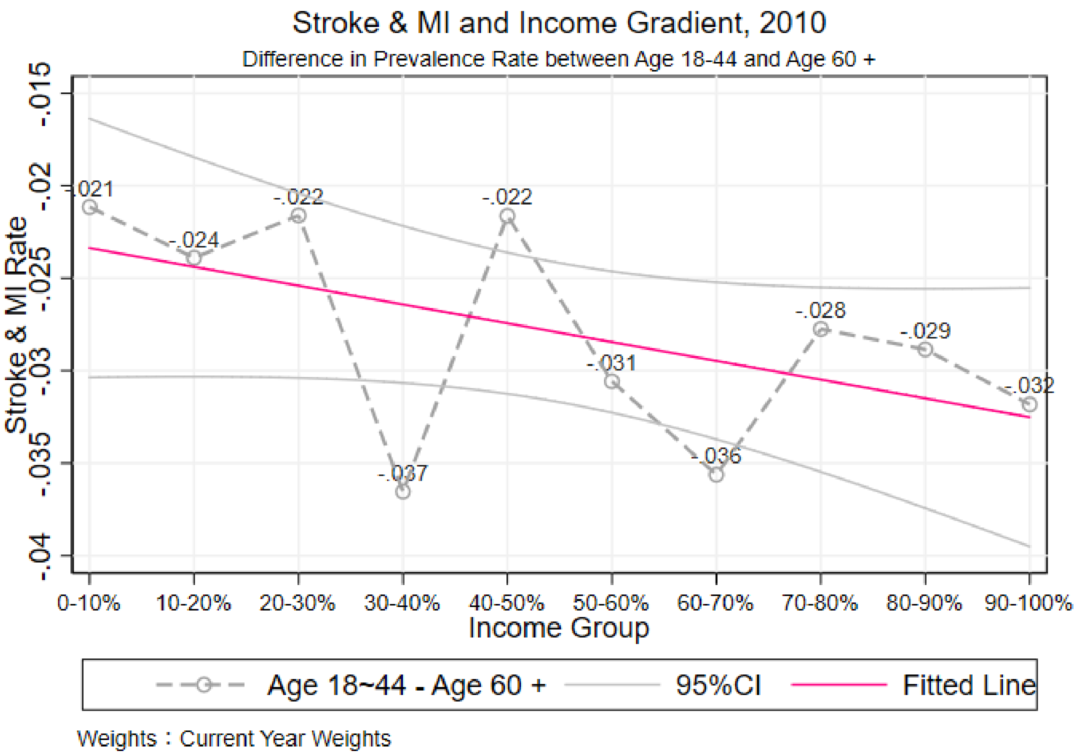

Figure A1 the Difference in Prevalence Rate between Age Group

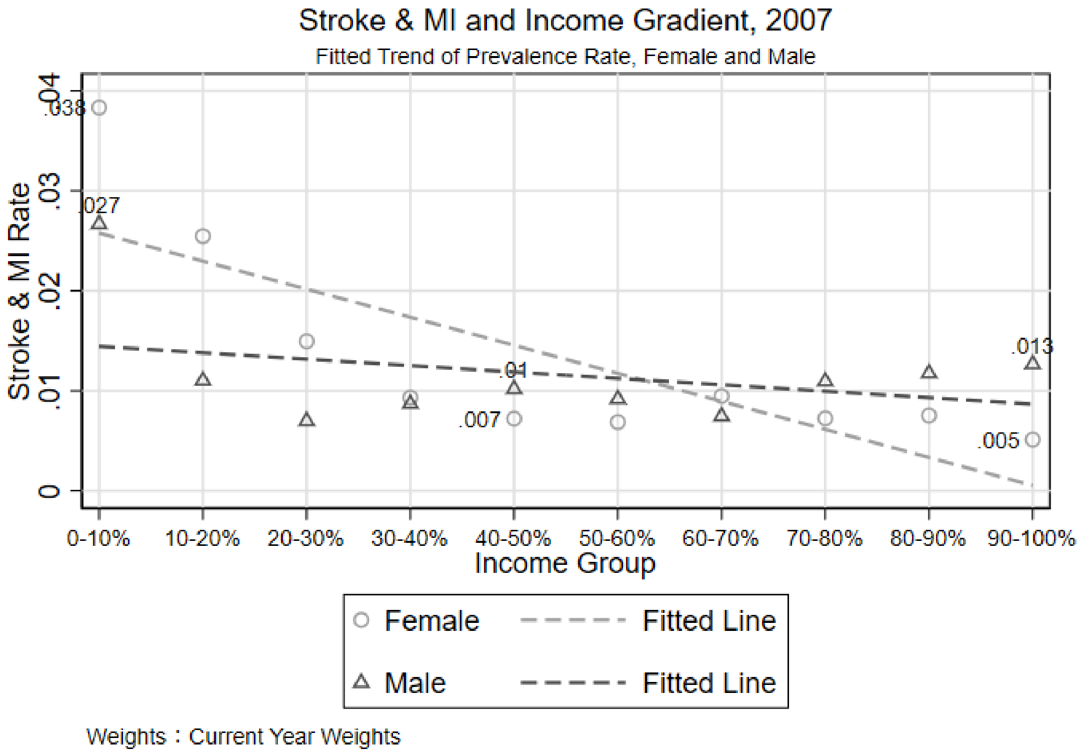

(a)

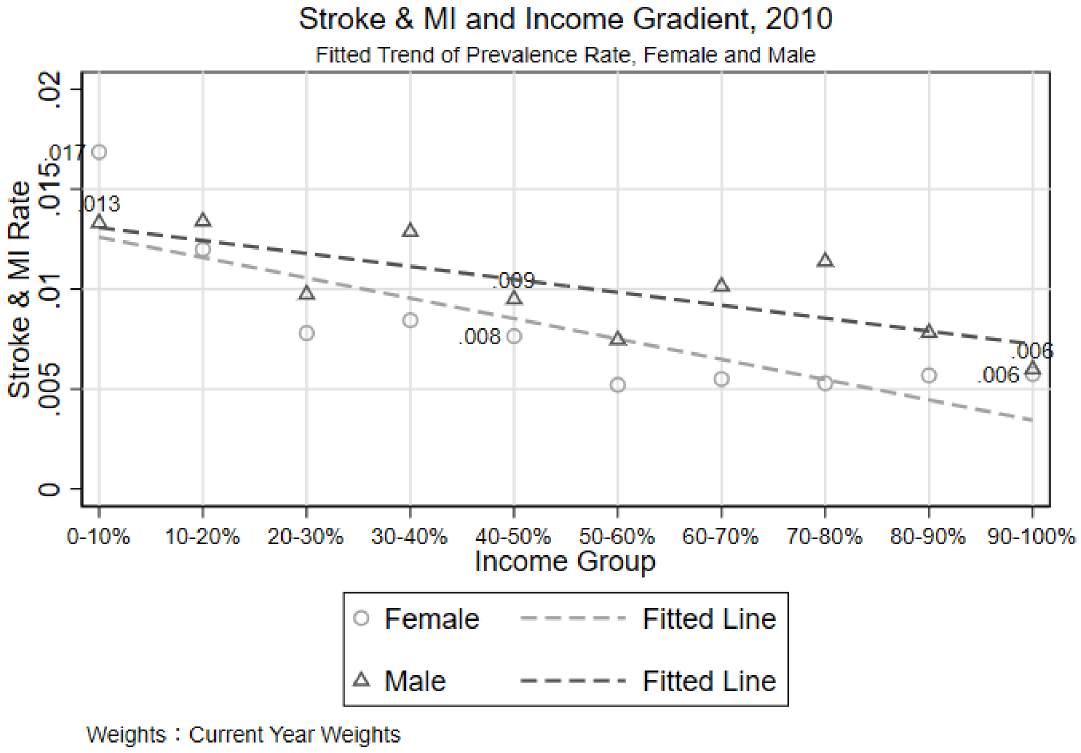

(b)

Figure A2 the Trend in Prevalence Rate of Male and Female Population

Note: The income gradient categorized based on overall sample, and in each income group we detected the prevalence rate in male and female separately.

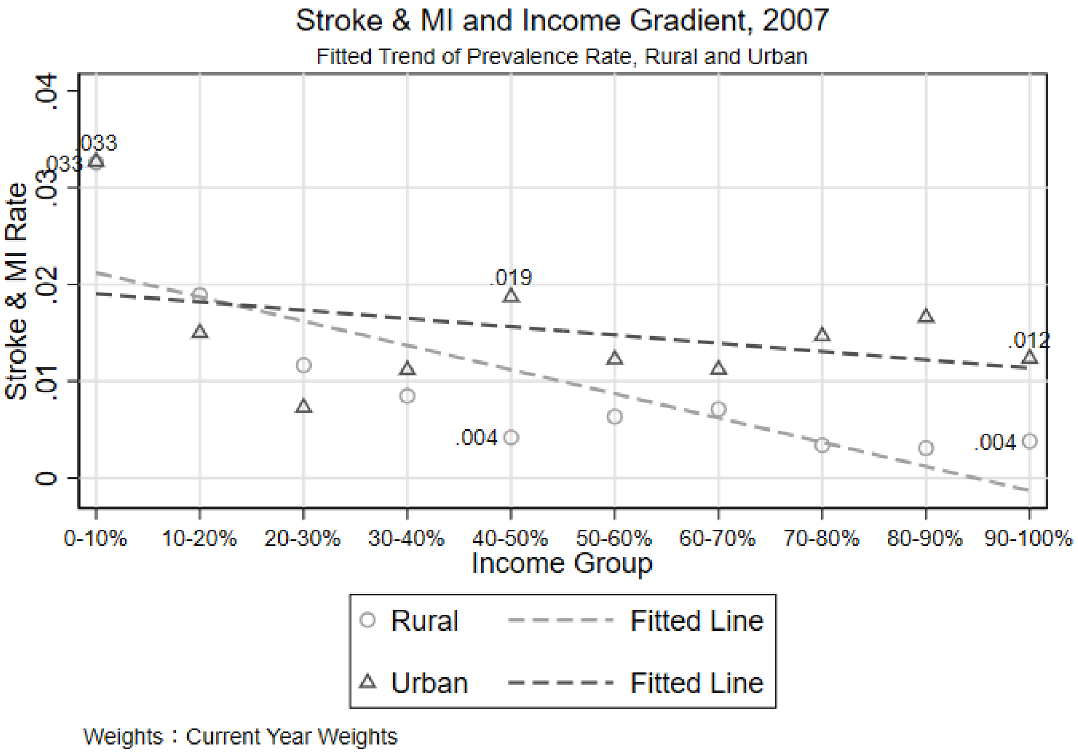

(a)

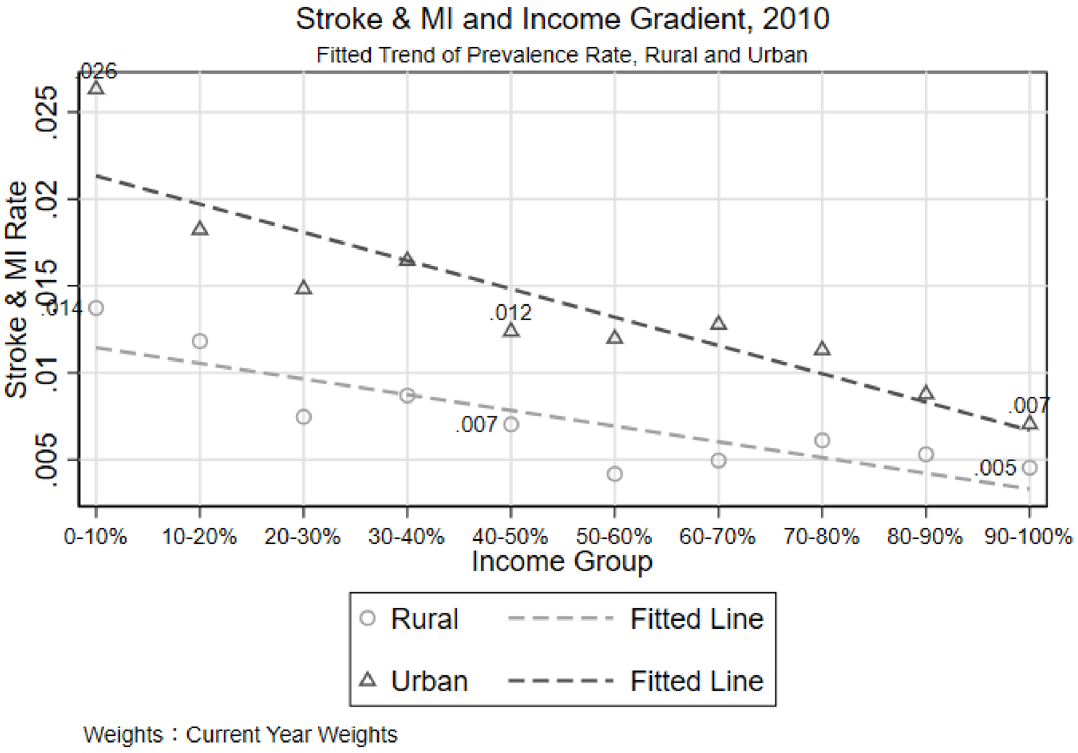

(b)

Figure A3 the Trend in Prevalence Rate of Urban and Rural Population

Notes: The income gradient categorized based on overall sample, and in each income group we detected the prevalence rate in male and female separately.

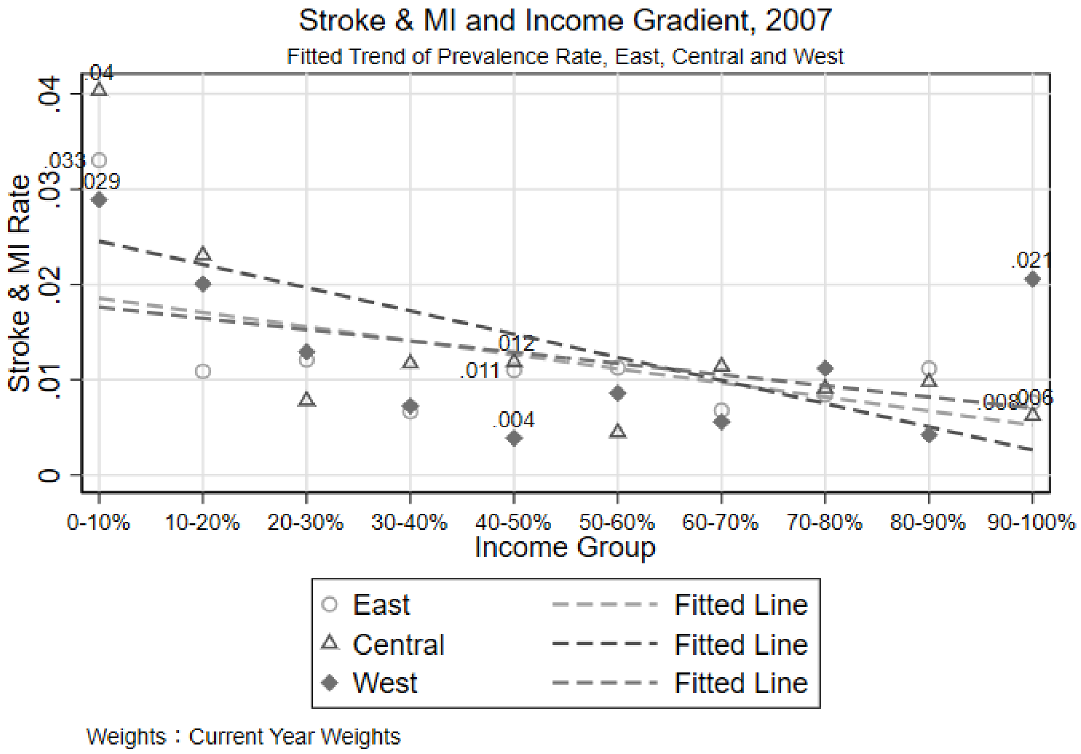

(a)

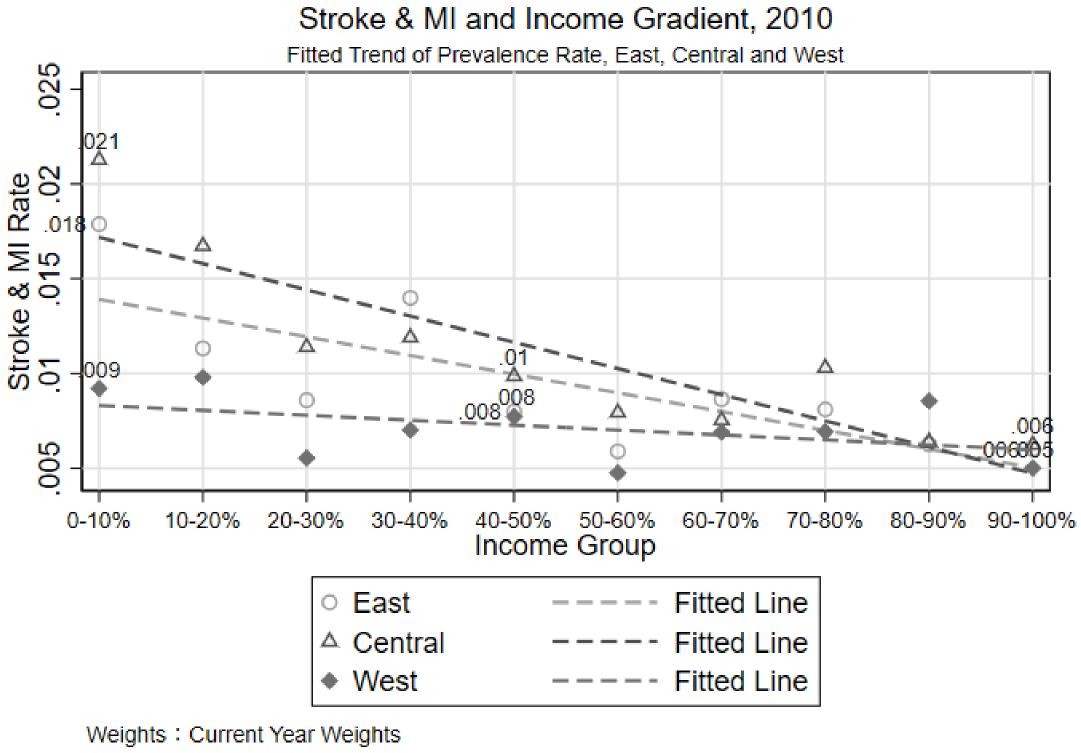

(b)

Figure A4 the Trend in Prevalence Rate among Eastern, Western and Central Regions

Notes: In Figure A4, we first grouped people into different income groups, then calculated the stroke and MI rate of sub-population grouped by regions in each income group.

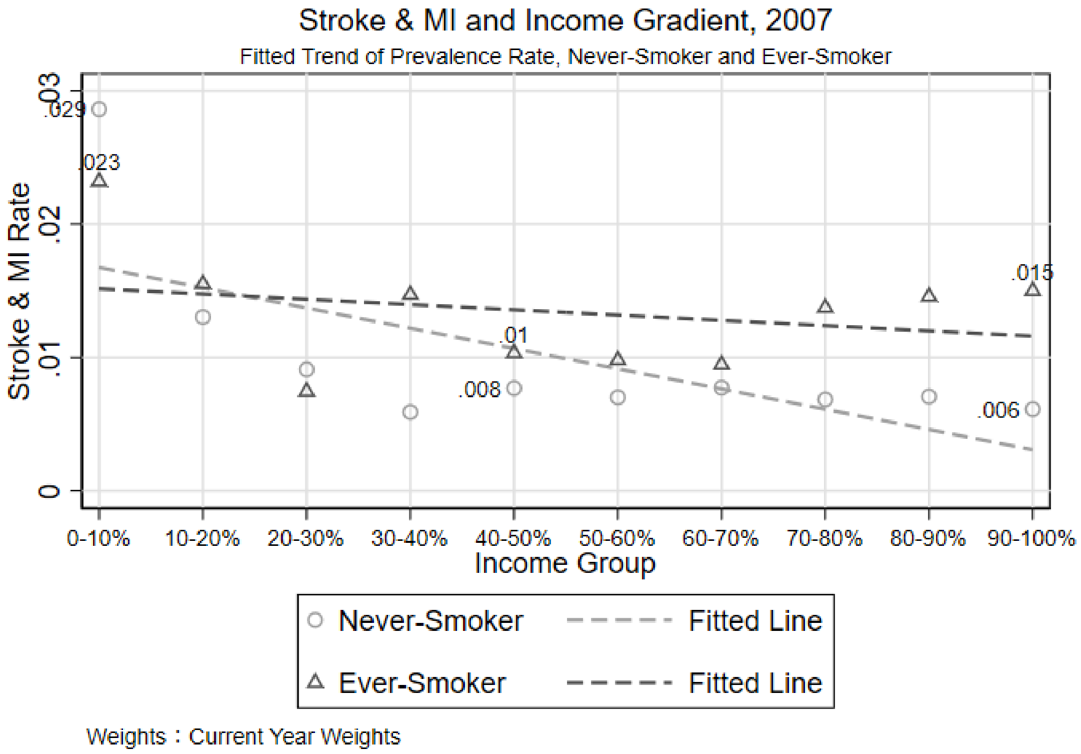

(a)

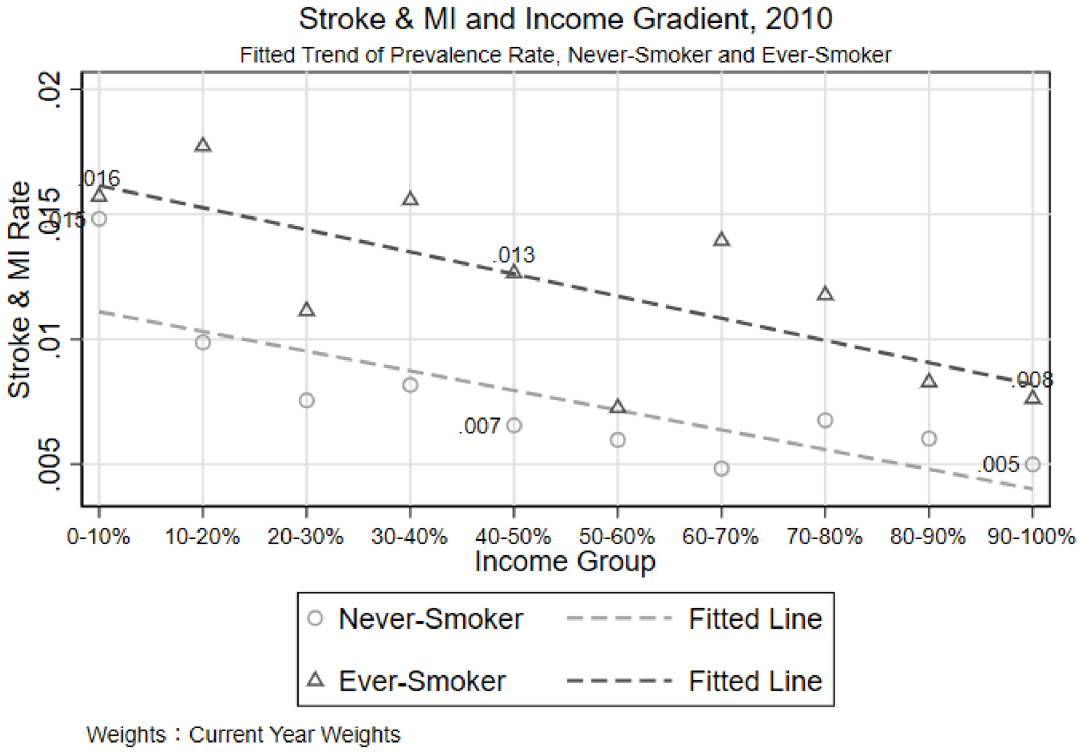

(b)

Figure A5 the Trend in Prevalence Rate between Smoking Behaviors

Notes: In Figure A5, we first grouped people into different income groups, then calculated the stroke and MI rate of sub-population grouped by smoking behaviors in each income group.

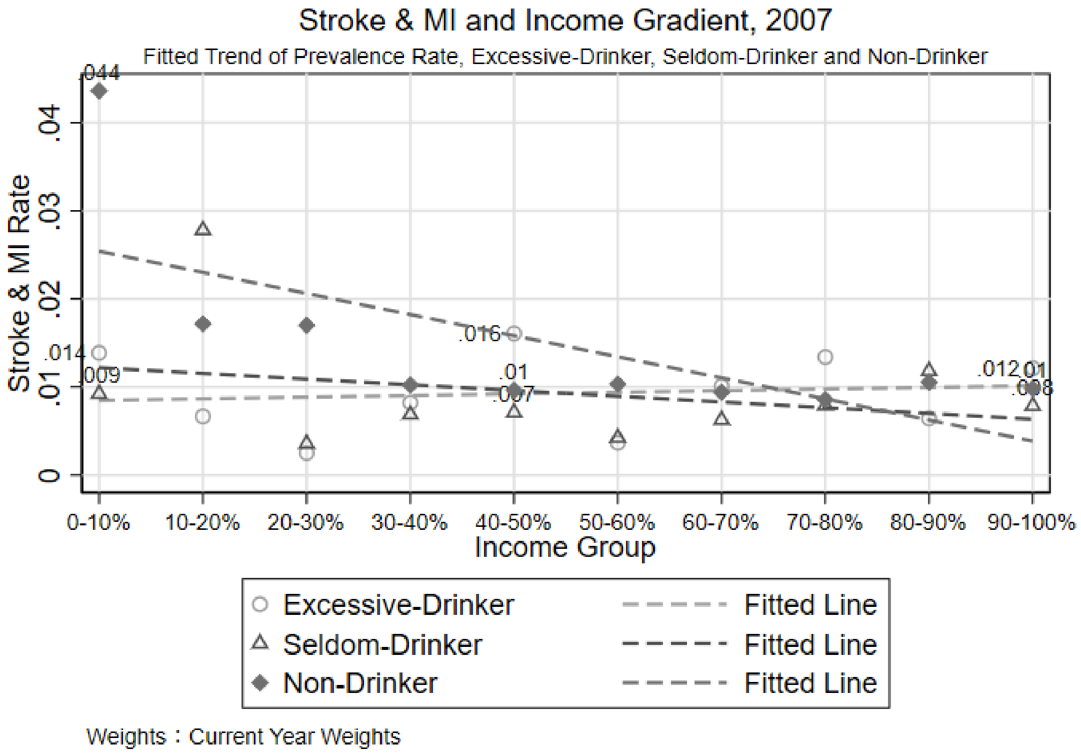

(a)

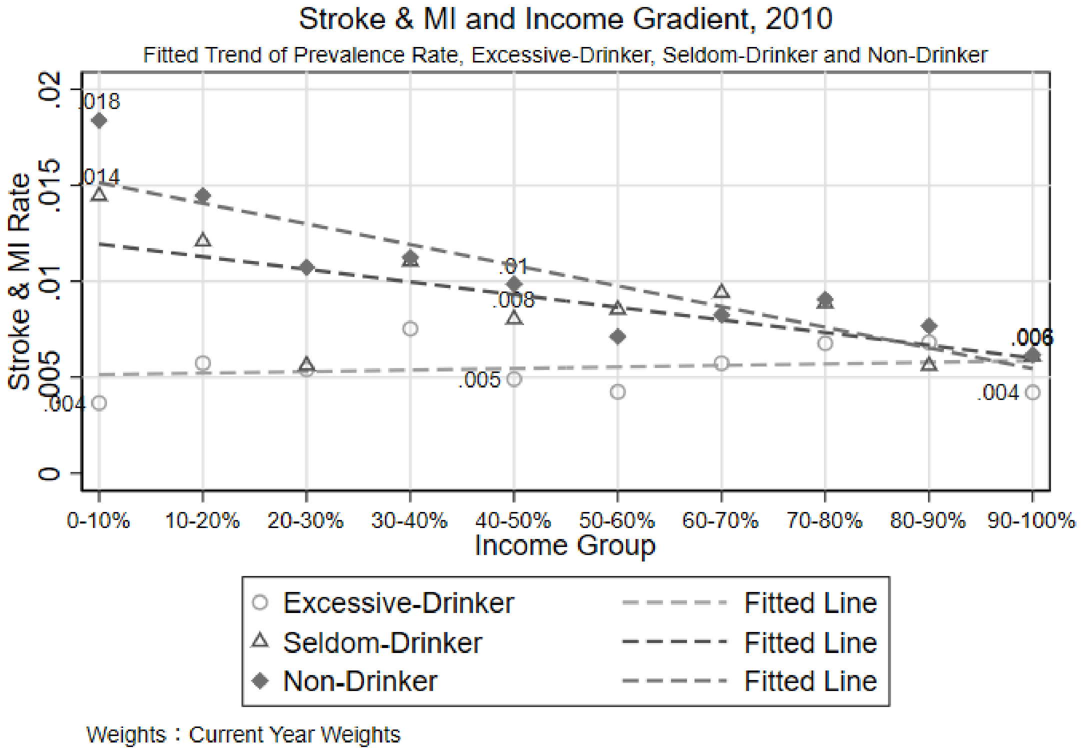

(b)

Figure A6 the Trend in Prevalence Rate between Drinking Behaviors

Notes: In Figure A6, we first grouped people into different income groups, then calculated the stroke and MI rate of sub-population grouped by drinking behaviors in each income group. People who had an excessive drinking behavior defined by CDC is identified as Excessive-Drinker, people who drink but without excessive drinking behaviors is categorized as Seldom-Drinker, people who do not drink at all is identified as Non-Drinker.
